# When time stands upright: STEARC effects along the vertical axis

**DOI:** 10.1007/s00426-022-01693-9

**Published:** 2022-06-19

**Authors:** Mario Dalmaso, Youval Schnapper, Michele Vicovaro

**Affiliations:** 1 Department of Developmental and Social Psychology, University of Padova, via Venezia 8, 35131 Padova, Italy; 2 Department of General Psychology, University of Padova, via Venezia 8, 35131 Padova, Italy

## Abstract

According to the spatial–temporal association of response codes (STEARC) effect, time can be spatially represented from left to right. However, exploration of a possible STEARC effect along the vertical axis has yielded mixed results. Here, in six experiments based on a novel paradigm, we systematically explored whether a STEARC effect could emerge when participants were asked to classify the actual temporal duration of a visual stimulus. Speeded manual responses were provided using a vertically oriented response box. Interestingly, although a top-to-bottom time representation emerged when only two temporal durations were employed, an inverted bottom-to-top time representation emerged when a denser set of temporal durations, arranged along a continuum, was used. Moreover, no STEARC effects emerged when participants classified the shapes of visual stimuli rather than their temporal duration. Finally, three additional experiments explored the STEARC effect along the horizontal axis, confirming that the paradigm we devised successfully replicated the standard left-to-right representation of time. These results provide supporting evidence for the notion that temporal durations can be mapped along the vertical axis, and that such mapping appears to be relatively flexible.

## Introduction

Time is one of the most crucial variables pervading our everyday activities, and it deeply shapes our interactions with both others and the surrounding environment. Starting with the ring of the alarm clock in the morning, which marks the beginning of a new day; then passing through appointments, meetings, and coincidences; and ending with the usual time we go to sleep, our entire life is a rich time-based stream of events. Hence, it is not surprising that research efforts have been made to investigate the concept of time and its impact on both human cognition and behavior (for a review, see Grondin, 2010).

Unlike other physical dimensions, time cannot be directly perceived. For instance, we can easily identify the color of an object or, if taken in hand, we can feel its weight, but we cannot directly perceive how much time has elapsed since it appeared in our sight. Although the concept of time can be grasped with relative difficulty because of the lack of a dedicated sensory system, converging evidence indicates that time is often mapped onto the more concrete and experience-based concept of space (see Boroditsky, 2000). Time-related concepts such as *before* and *after*, or time durations such as *short* and *long*, would be represented as if they were placed at the two ends of a hypothetical spatially oriented line (for reviews, see Bender & Beller, 2014; Bonato et al., 2012; Núñez & Cooperrider, 2013). In Western cultures this line can unfold along a horizontal left-to-right spatial direction, with *before* and *short* represented on the left side of the space, and *after* and *long* on the right (Santiago et al., 2007; Vallesi et al., 2008; Weger & Pratt, 2008; Zhao et al., 2018). On the contrary, a right-to-left representation of time has been documented among Arabic- and Hebrew-speaking people, at least under some circumstances (e.g., Fuhrman & Boroditsky, 2010; Ouellet et al., 2010; Tversky et al., 1991; but see Vallesi et al., 2014). These results seem to suggest that cultural habits such as reading and writing direction deeply shape the nature of space–time associations (see also Pitt & Casasanto, 2020). Furthermore, other studies have shown that time can be mapped onto the sagittal axis (i.e., the posterior–anterior axis), with *before* and *short* represented as behind the viewer, and *after* and *long* in front, likely caused by humans’ habitual walking direction (i.e., *forward*; Bender & Beller, 2014; Boroditsky, 2000; Eikmeier et al., 2013; Kolesari & Carlson, 2018; Rinaldi et al., 2016; Torralbo et al., 2006). The mapping of time onto space is therefore influenced by both cultural and embodied factors.

### Horizontal spatial–temporal association of response codes effect

The possible relationship between time and space has been explored using a variety of experimental paradigms (see Bender & Beller, 2014). Most studies have adopted tasks in which temporal-related dimensions were classified by using response keys placed at the two ends of a spatial reference system, such as the horizontal plane (i.e., a left-side vs. right-side response key). On the one hand, these temporal judgement tasks can be based on the discrimination of the actual passage of time, requiring a quantitative estimation of the length of the temporal duration of a stimulus, a time concept which is also known as *temporal span* (Núñez & Cooperrider, 2013). For instance, Vallesi et al. (2008) asked Western participants to classify the time duration of a central visual stimulus (a yellow cross) as either short (e.g., lasting 1 s) or long (e.g., lasting 3 s) using left and right keys. Short and long durations were responded to more quickly with the left-side and the right-side keys, respectively, than with the opposite mapping (i.e., short-right/long-left; see also Vallesi et al., 2011, 2014). On the other hand, temporal judgement tasks can require processing the temporal information associated with symbolic stimuli describing either past versus future events or earlier versus later relationships (time concepts which are also known as *deictic time* and *sequence time*, respectively; Núñez & Cooperrider, 2013). For instance, Weger and Pratt (2008) asked Western participants to categorize a name as belonging to an actor who was popular either before or after they were born (e.g., Charlie Chaplin vs. Kate Winslet). Responses were given using left- and right-side keys. In line with Vallesi et al. (2008), faster responses emerged when *before* was associated with the left-side key and *after* with the right-side key, compared with the condition in which the before-right/after-left mapping was adopted.

The left-to-right spatial mapping of time appears to be particularly robust and reliable, and has emerged in response to a variety of different time-related stimuli such as auditory tones of varying time durations (Ishihara et al., 2008), past- and future-related words (e.g., “he spoke” vs. “they will think”; e.g., Grasso et al., 2021; Ouellet et al., 2010; Santiago & Lakens, 2015; Santiago et al., 2007), numerical measures of time (e.g., 1 h vs. 1 day; Zhao et al., 2018), and pictures depicting temporally ordered actions or events (e.g., ancient vs. futuristic cities; Miles et al., 2011; a fruit being eaten or a young person getting old; Boroditsky et al., 2011; Fuhrman & Boroditsky, 2010; Kolesari & Carlson, 2018; but see Dalmaso & Vicovaro, 2021).

The way time can be mapped onto space closely resembles what generally emerged from the numerical cognition literature. In this regard, the well-known spatial–numerical association of response codes (SNARC) effect (Dehaene et al., 1993) refers to the phenomenon by which relatively small (typically digits 1–4) and large (typically digits 6–9) numbers are responded to more quickly with left- and right-side response keys, respectively, than when the opposite mapping is adopted (small-right/big-left). The SNARC effect can emerge both when the number magnitude is treated explicitly (i.e., digits have to be categorized as either lesser or greater than the reference number 5) or implicitly (i.e., digits have to be categorized as either even or odd). This indicates an automatic processing of number magnitude (Dehaene et al., 1993). The SNARC effect supports the idea that number magnitudes are disposed along a physical continuum, where small numbers are represented on the left and large numbers are represented on the right. This hypothetical spatial representation is often referred to as the “mental number line” (Moyer & Landauer, 1967). Like the direction of the mental time line, the direction of the mental number line also appears to be shaped by reading and writing direction (Fischer et al., 2009; Shaki et al., 2009). Hence, the overlapping between mental representations of numbers and time appears to be very tight (but see Pitt & Casasanto, 2020). This is also reflected in the similarity between the nomenclatures adopted in the numerical (SNARC, mental number line) and temporal domains (spatial–temporal association of response codes [STEARC], mental time line).

In recent years, SNARC-like effects have been documented even for nonnumerical magnitudes other than time, such as size (e.g., Prpic et al., 2020; Ren et al., 2011; Sellaro et al., 2015), weight (Dalmaso & Vicovaro, 2019), or luminance (Fumarola et al., 2014), confirming that the general notions of less and more are generally associated with the left and right sides of physical space, respectively (see also Macnamara et al., 2018, for a review). Similarly to the SNARC effect, SNARC-like effects can also be detected when the considered magnitude is treated implicitly (Fumarola et al., 2014; Sellaro et al., 2015).

### Theories of space–magnitude associations

The similarities between the SNARC effect and SNARC-like effects (including the STEARC effect) have inspired many different theories and models aimed at explaining the relationship between space and magnitude. In the present context, for the sake of clarity, we will briefly discuss what are likely the three main theories on this topic: the so-called “a theory of magnitude” (ATOM; Walsh, 2003, 2015), the polarity correspondence model (Proctor & Cho, 2006), and the theory according to which space–magnitude associations would be shaped by reading/writing direction (e.g., Dehaene et al., 1993).

According to ATOM, specific mechanisms for processing time, numbers, and space may originate from a general common system devoted to processing magnitudes. The existence of this common underlying mechanism would explain the similarities among the spatial representations of time and numbers, as well as the partial overlap between the neural structures devoted to processing time, numbers, and space (Toomarian & Hubbard, 2018).

A radically different theoretical account is provided by the polarity correspondence model (Proctor & Cho, 2006), according to which both space and magnitude can be represented as polarized concepts: The left/rear/lower parts of the space would be coded as negative, whereas the right/front/higher parts of the space as positive. In a similar vein, small magnitudes (e.g., smaller numbers, shorter time durations) would be coded as negative and large magnitudes (e.g., greater numbers, longer time durations) as positive. According to the polarity correspondence principle, identical polarities should be associated with each other: For instance, the left side of space (negative polarity) should be preferentially associated with shorter time intervals (negative polarity), and the right side of space (positive polarity) should be preferentially associated with longer time intervals (positive polarity). In other words, according to this model, both space–time and space–number relationships are the result of a general mechanism of conceptual correspondence.

Lastly, various authors have suggested that space–magnitude associations would be shaped by reading/writing direction (e.g., Dehaene et al., 1993). Empirical support for this hypothesis comes, for instance, from a cross-cultural study by Shaki et al. (2009), in which a left-to-right SNARC effect emerged for Canadians (who read and write words and numbers from left to right), a reversed right-to-left SNARC effect emerged for Palestinians (who read and write words and numbers from right to left), and no evidence of a SNARC effect emerged for Israelis (who read words from right to left and numbers from left to right). This suggests that space–magnitude associations result from the interaction between the general reading/writing direction and the specific reading/writing direction for numbers.

### The flexibility of space–magnitude representations and its theoretical implications

An intriguing characteristic of spatial representations of magnitudes is that besides being shaped by long-term cultural habits (e.g., reading/writing direction), they can also be flexible, according to a variety of contextual factors. For instance, both the way in which instructions are provided to participants and the presence of task-irrelevant digits placed in SNARC-incompatible locations can affect the strength and direction of the SNARC effect (Bächtold et al., 1998; Fischer et al., 2010; Lindemann et al., 2008). For instance, in Bächtold et al. (1998), a standard left-to-right SNARC effect emerged when participants were asked to imagine the digits from 1 to 11 as numerical distances placed on a classic ruler—in which the number magnitude follows a left-to-right direction—whereas an inverted right-to-left SNARC effect emerged when the same digits were imagined like the hours represented on an analogue clock with moving hands (on a clock face, the relatively small digits 1–5 are placed on the right side of the clock, and the relatively large digits 7–11 on the left side of the clock). Similarly, Lindemann et al. (2008) found that the standard left-to-right SNARC effect vanished when target numbers were presented within a sequence of decreasing numbers, following a right-to-left displacement. Hence, spatial representations of numbers appear to be contextually malleable mental constructs (see also Gevers et al., 2006; Santens & Gevers, 2008; Pitt & Casasanto, 2020).

More relevant for the present work, spatial representations of time also appear to be flexible. Of particular interest is the study of Torralbo et al. (2006), who conducted two experiments in which a schematic human head, oriented leftwards or rightwards, appeared at the center of the screen together with a text balloon that could appear either on the left or the right side of the head. The position of the balloon could be interpreted either as placed on the left or right side of the physical space (i.e., the egocentric frame of reference), or as placed in front of or behind the schematic head (i.e., the allocentric frame of reference). Critically, on each trial, the balloon contained either a past- or a future-related sentence (e.g., “he spoke” vs. “they will think”). In both experiments, participants were asked to report whether the individual represented by the schematic head was thinking about past or future events. Participants provided verbal responses in Experiment 1 and manual responses collected using lateralized (left vs. right) response keys in Experiment 2. Two distinct patterns of results emerged from the experiments. A sagittal back-to-front representation of time (based on the perspective of the schematic head) appeared in Experiment 1, and a horizontal left-to-right representation in Experiment 2. According to Torralbo et al. (2006), a horizontal left-to-right representation of time emerged only in Experiment 2 because, in that case, participants had to respond using lateralized response keys, which likely made the horizontal dimension of the space particularly salient. According to this interpretation, different space–time mappings may emerge in different contexts depending on the saliency of the available dimensions of space. The flexibility of horizontal spatial representations of time is also highlighted by the results of Casasanto and Bottini (2014; Experiment 1), in which Dutch participants classified past- and future-related sentences using lateralized response keys. A standard left-to-right representation emerged for sentences written in standard orthography, whereas a reversed right-to-left representation emerged for sentences written in mirror-reversed orthography. Therefore, reading direction appears to shape the direction of the mental time line (see also Pitt & Casasanto, 2020; but see Beracci et al., 2021a, 2021b).

From a theoretical viewpoint, various authors have suggested that the flexibility characterizing both space–time and space–number representations might reflect the crucial role of working memory in constructing these representations (e.g., Fischer, 2006; Herrera et al., 2008; Torralbo et al., 2006; van Dijck & Fias, 2011; van Dijck et al., 2009). In the case of space–time representations, Torralbo et al. (2006) suggested that in experiments in which participants are required to classify time-related stimuli, working memory would try to achieve the most globally coherent spatial representation of the stimuli. Mapping time onto the most salient dimension of space would then maximize the global coherence of the space–time representation, facilitating the encoding of the stimuli. In other words, representing the time-related stimuli onto the most salient dimension of space facilitates the participant’s task. When no specific dimension of space is made salient by the context-specific features of the task, working memory may rely on representations shaped by long-term habits like writing or walking direction, which are stored in long-term memory.

### Mental representation of magnitudes on the vertical axis

Some studies suggest that the spatial representation of magnitudes can also operate along the vertical axis. Within this spatial framework, “less” appears to be associated with the bottom part of the space, and “more” with the top part of the space. This bottom-to-top representation has been documented both for the SNARC effect (e.g., Müller & Schwarz, 2007; Schwarz & Keus, 2004; see also Ito & Hatta, 2004) and for SNARC-like effects evoked by nonnumerical quantities, such as pitch (Rusconi et al., 2006), loudness (Bruzzi et al., 2017), and weight (Vicovaro & Dalmaso, 2021). The bottom-to-top representation of quantities might reflect some constraints of our physical world that we often experience in everyday life contexts, such as when we stack objects on top of one another (the more objects we stack, the more they “go up”) or when we fill a glass with a liquid (the more liquid poured, the higher the level reached in the glass).

According to some authors (e.g., Myachykov et al., 2014), this strong relationship linking increasing quantities with a bottom-to-top vector would make vertical representations of magnitudes much more stable and universal compared with horizontal ones, leading to the common expression that “more is up” (see also Shaki & Fischer, 2012, 2018). Nevertheless, in partial contrast with this claim, some other studies have shown that even the vertical spatial representations of magnitudes can be flexible. Indeed—and similarly to what has been observed for the horizontal dimension—it has been found that the direction of the vertical SNARC effect can be affected by task-specific instructions, such as imagining numbers as either floors in a building (which are typically listed from bottom to top) or as depth levels of a swimming pool, which are instead arranged from top to bottom (Holmes & Lourenco, 2012). Moreover, the direction of the vertical SNARC effect also appears to be affected by which effectors are used to respond to small and large numbers. Indeed, the bottom-to-top representation that typically emerges when only manual responses are allowed (e.g., Müller & Schwarz, 2007) can be reversed when responses are provided using hands (associated with faster responses to smaller numbers) and feet (associated with faster responses to larger numbers; see Hartmann et al., 2014a, 2014b).

As for the vertical representation of nonnumerical magnitudes, Vicovaro and Dalmaso (2021) observed that the bottom-to-top representation of weight that emerged for word stimuli conveying abstract concepts of weight (e.g., the words paper vs. iron) was inverted for stimuli associated with the weight of concrete items which had been manually weighed by the participants before the experiment. This likely reflects an embodied spatial representation of weight shaped by the force of gravity, which acts from top to bottom. Overall, the results of these studies highlight the flexibility of vertical spatial representations of both numerical and nonnumerical magnitudes.

### A vertical STEARC effect?

Although an increasing number of studies indicate the existence of vertical SNARC-like effects for nonnumerical quantities, the vertical representation of time appears to be not so well defined (at least among Western individuals).

The first study on this topic was based on a quantitative estimation of the actual length of the temporal duration of a stimulus (Ishihara et al., 2008). German participants were tested in two experiments in which seven acoustic stimuli, interleaved by a fixed inter-onset interval (IOI) of 500 ms, were delivered. Then, an eighth acoustic stimulus (i.e., the probe) was provided as well, but it could be presented either earlier (285 ms) or later (715 ms) in time than the 500-ms IOI. Participants were asked to classify the timing of the probe as earlier or later than the standard 500-ms IOI by pressing one of the two keys on a response box. In Experiment 1, in which a horizontal STEARC effect was tested, the response box was placed horizontally, so that one key was on the left and the other on the right. In Experiment 2, in which a vertical STEARC effect was tested, the response box was rotated 90° on the horizontal plane, so that one key appeared to be placed “above” the other key. Although a horizontal left-to-right STEARC effect emerged in Experiment 1, Experiment 2 failed to provide evidence for a reliable vertical STEARC effect. However, it is important to note that the response keys in Experiment 2 operated along the sagittal plane (i.e., near/far to the participant) rather than on a truly vertical axis. This means that the two response buttons were not actually placed along the appropriate physical dimension, which is crucial to investigate both SNARC and SNARC-like effects properly (Winter et al., 2015). Nevertheless, support for the lack of a vertical STEARC effect among Western individuals also emerged in Kolesari and Carlson (2018), who asked English speakers to classify central pictures showing *earlier* or *later* events by pressing vertically oriented handheld switches that were kept in a close position (i.e., both hands in front of the participant’s chest), in a medium distance position (i.e., one hand in front of the chest and one below the chair), or in a far position (i.e., one hand above the head and one below the chair). In none of these conditions, a vertical STEARC effect emerged.

Other studies found vertical STEARC effects by employing stimuli that increased the saliency of the vertical dimension of space. In Casasanto and Bottini (2014; Experiment 2), Dutch participants classified past- and future-related sentences using vertically aligned response keys. No evidence of a vertical representation of time emerged when the sentences were presented in standard left-to-right orthography, but a bottom-to-top representation emerged when the sentences were presented rotated 90° (i.e., written from bottom to top of the screen), and the opposite top-to-bottom representation emerged when they were rotated 270° (i.e., written from top to bottom). More recently, Beracci et al. (2021a, 2021b) presented Italian participants with a central visual reference lasting 400 ms, followed by a visual target lasting 200, 300, 500, or 600 ms. Target position varied along the vertical axis of the screen (i.e., bottom, center, or top). Participants classified target durations as shorter or longer than the reference by pressing two “vertically aligned” keys on a horizontal keyboard (responses actually operated along the sagittal plane, as in Ishihara et al., 2008). Shorter durations were responded to faster with the “bottom” key than with the “top” key, and the opposite was true for longer durations.

Additional support for a vertical representation of time among Westerners emerged from studies in which time was treated as an implicit dimension. In Hartmann et al. (2014a, 2014b), German-speaking participants were asked to think about their past and future, while spontaneous eye movements were recorded. More rightwards and upwards eye movements emerged when thinking about the future than the past, in line with a diagonal bottom-left/top-right spatial representation of time (for similar results see also Stocker et al., 2016). Recently, in Topić et al. (2021) Croatian-speaking participants were first presented with a central target lasting for a variable duration, and then with a spatially oriented array of items that could contain the target. Participants detected the presence of the target in the array. Unlike Hartmann et al. (2014a, 2014b) and Stocker et al. (2016), Topić et al. (2021) showed that long and short target durations facilitated its detection in the lower and upper half of the search array, respectively. However, the relationship between the results of these studies and the spatial representation of time is not entirely clear. Indeed, as highlighted by Stocker et al. (2016), it cannot be excluded that a pattern of eye movements along a bottom-left/top-right diagonal may actually reflect a sagittal back-to-front spatial representation, rather than a genuine vertical representation. Moreover, despite the well-documented left-to-right representation of time, this did not emerge in Topić et al. (2021) when the targets appeared on the left or on the right side of the search array.

Other studies found supporting evidence for a vertical representation of time only when cultural differences were considered. Boroditsky et al. (2011) presented English and Mandarin speakers with pictures depicting two temporally ordered actions or events (i.e., two photos of Woody Allen at an early or late time in his life). The participants were asked to report whether the second picture referred to an earlier or later action or event relative to the first picture. Responses were collected through horizontally or vertically aligned response keys. Although a similar left-to-right STEARC effect occurred in both groups evidence for a vertical STEARC effect emerged only among Mandarin speakers, who responded more quickly when *earlier* was associated with a top key and *later* was associated with a bottom key (see also Boroditsky, 2001; Miles et al., 2011; Fuhrman et al., 2011; Yang & Sun, 2016; but see Chen, 2007; January & Kako, 2007). This top-early/bottom-late space–time mapping may reflect the presence of specific metaphors in the Mandarin language that link the concepts early and late to the upper and lower parts of space, respectively, whereas such metaphors are basically missing in English (Sun & Zhang, 2021), as well as in many other languages (Italian included). The idea that language could shape time representation has found further support in Hendricks and Boroditsky (2017), in which a vertical representation of time actually emerged even among English speakers, but only when, in an initial phase, they were taught new metaphors of time created ad hoc for experimental purposes (e.g., “Tuesday is above Wednesday” or “breakfast is above dinner”).

It is important to note that the aforementioned studies describing a vertical representation of time among Mandarin speakers required participants to retrieve the temporal information associated with a given symbolic stimulus, such as a word or a picture (deictic and sequence time; see Núñez & Cooperrider, 2013), and therefore both linguistic and semantic components of the stimuli were highly involved in the task. This feature may have contributed to the linguistic and cultural differences that emerged in those studies. Because, to our knowledge, the studies of Ishihara et al. (2008) and Beracci et al. (2021a, 2021b) represent the only attempts to explore the vertical representation of time by requiring estimates of actual temporal durations (temporal span; see Núñez & Cooperrider, 2013), we believe that more work is needed on that front.

### Implications of the (lack of) vertical STEARC effect for theories of space–magnitude associations

Under a theoretical perspective, the current theories of space–magnitude representations cannot easily account for the lack of a clear and reliable vertical STEARC effect among Westerners. On the one hand, ATOM (Walsh, 2003, 2015) postulates that the spatial representations of both numbers and time are similar to each other. Therefore, the consistent bottom-to-top vertical representation that is typically observed for numbers, should emerge for time-related stimuli as well (i.e., short-bottom/long-top). On the other hand, according to the polarity correspondence model (Proctor & Cho, 2006), the upper part of space and longer time durations should be coded as positive polarities, whereas the lower part of space and shorter time durations should be coded as negative polarities. In line with ATOM, the polarity correspondence model should also predict short-bottom/long-top mapping. Hence, in the perspective of both ATOM and the polarity correspondence model, the lack of a defined vertical STEARC effect among Westerners is a special case requiring specific explanation, which, to the best of our knowledge, has not been provided yet.

Besides being inconsistent with ATOM and the polarity correspondence model, the lack of a vertical STEARC effect for Western participants is also inconsistent with the results of previous studies showing that spatial representations of time are affected by reading/writing direction (e.g., Bergen & Chan Lau, 2012; Casasanto & Bottini, 2014; Fuhrman & Boroditsky, 2010; Ouellet et al., 2010; Pitt & Casasanto, 2020; Tversky et al., 1991). In Western cultures, reading and writing proceed not only from left to right, but also from top to bottom (you probably started reading this page from the top). This vertical arrangement is even more perceptually salient in Western comics, where the events of a story are typically clustered within geometrical panels that flow from the top to the bottom part of the page, and in calendars, where the days of the month start from the top part of the page.^1^ Hence, according to the hypothesis that reading/writing habits affect the spatial representation of time, Western participants could also exhibit a vertical STEARC effect in which shorter time durations and earlier events should be represented as *up*, and longer time durations and later events should be represented as *down*.

In sum, on the one hand, both ATOM and the polarity correspondence model predict a bottom-to-top representation of time-related stimuli; on the other hand, the opposite mapping is expected to emerge if one assumes that spatial representations of time are shaped by one’s reading/writing direction. However, none of these hypotheses is consistent with the absence of a defined vertical STEARC effect in Western participants.

### The present study

Our work was aimed at revealing the presence of a STEARC effect along the vertical axis in Western participants across six experiments. Inspired by Vallesi et al. (2008), we developed a novel task requiring participants to classify the actual temporal duration (temporal span; see Núñez & Cooperrider, 2013) of a central visual stimulus (i.e., a square vs. a diamond) that remained on a computer screen for a variable temporal duration. In doing so, we aimed to test the two aforementioned alternative hypotheses—that deriving from ATOM and polarity correspondence model versus from reading/writing direction, respectively—concerning the representation of time along the vertical axis.

A systematic and comprehensive approach was adopted. Participants were presented either with only two temporal durations (100 or 900 ms) requiring classification as either short or long (Experiment 1a), or with several temporal durations (100–400 ms and 600–900 ms; 100-ms step) requiring classification as either shorter or longer than a 500-ms time reference (Experiment 2a). We also explored whether the STEARC effect could emerge even when the time duration was treated as an implicit dimension—that is, when participants were asked to classify the shape, rather that the duration, of target stimuli (Experiments 1b and 2b)—and the possible role of the time reference in shaping the STEARC effect (Experiments 3 and 4). Importantly, in Experiments 1a–4, we employed a response box with keys that were aligned along the vertical dimension, excluding potential confounds with other axes (sagittal) that had appeared in previous work (Beracci et al., 2021a, 2021b; Ishihara et al., 2008). Moreover, all the stimuli were presented at the center of the screen, thus preventing that possible STEARC effects could be influenced by the vertical arrangement of the stimuli (Beracci et al., 2021a; Beracci et al., 2021b; Casasanto & Bottini, 2014). In addition, we conducted three further experiments (Experiments 5–7; see the Appendix) employing horizontally placed response keys, with the aim to confirm that the experimental paradigm we devised was suitable to reveal the well-documented left-to-right STEARC effect (Vallesi et al., 2008).

## Experiment 1a

In this first experiment, participants were asked to classify a central visual stimulus as either *short* (100 ms) or *long* (900 ms), following a similar approach as that described in Vallesi et al. (2008, Experiment 1). Speeded manual responses were collected using two vertically aligned response keys. Here, and in all the following experiments, we have fully described how the sample size was established, as well as the experimental manipulations, the collected measures, and the data exclusion criteria (see, e.g., Simmons et al., 2011).

### Participants

Sample size was determined by following the guidelines for linear mixed-effects models including subjects and items as random factors (Brysbaert & Stevens, 2018). Hence, a minimum of 1600 observations per condition were necessary to reach adequate statistical power in the case of the small effect sizes that generally characterize reaction time studies. We planned to collect 68 trials per condition for each participant, so a minimum of 24 participants were necessary. We stopped at *N* = 28 (Mean age = 20 years, *SD* = 1.79, 10 males) for convenience at the end of a booking session, to achieve adequate statistical power. Two participants declared that they were left-handed. Manual preference was further evaluated with the 10-item Edinburgh Handedness Inventory (EHI; Oldfield, 1971). This provides a continuous score that can vary from −100 (strongly left-handed) to 100 (strongly right-handed). Our sample had a mean EHI score of 48 (*SD* = 41.15; range: −100 to 100). More precisely, two participants had negative EHI scores (−44; −100), one participant had a null EHI score (0), and the remaining participants had positive EHI scores (from 23 to 100). The study was approved by the Ethics Committee for Psychological Research at the University of Padova (protocol number 1882) and conducted in accordance with the Declaration of Helsinki. All participants were naïve about the purpose of the study, had normal or corrected-to-normal vision, and provided written informed consent.

### Apparatus

**Fig. 1:**
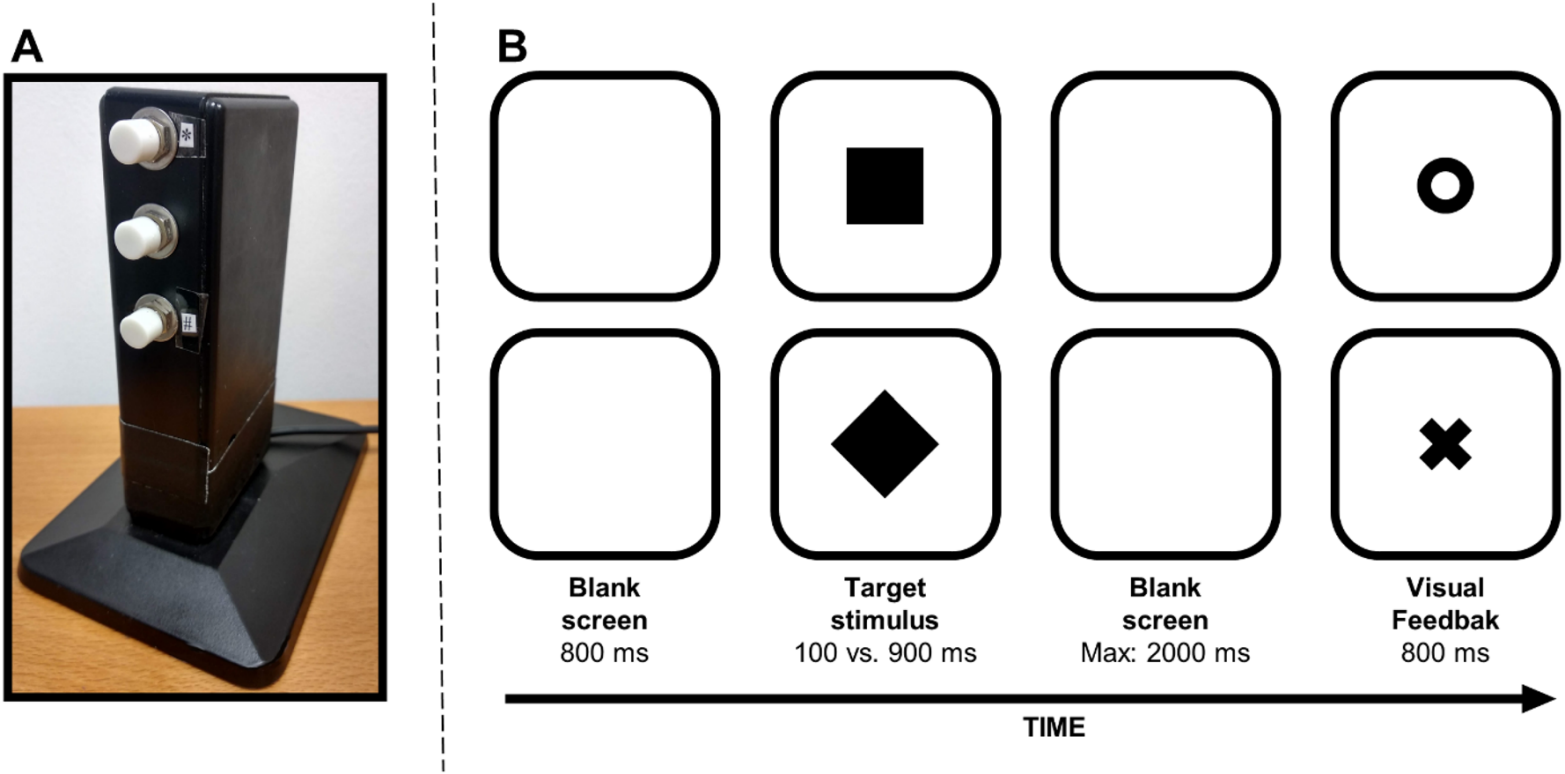
Vertical response box (**A**) and examples of trials (**B**). Upper panel = square shape, lower panel = diamond shape. O = correct response, X = incorrect response. Stimuli not drawn to scale

E-Prime 2 (Psychology Software Tools, Pittsburgh, PA) was used to generate the experiment. A PC monitor (1600 × 1200 pixels; 75 Hz), placed 57 cm from the participant, was used to present the stimuli. A custom-made response box (see Fig. 1a), placed centrally with respect to the screen, was used to collect manual responses. The response box keys were vertically arranged, which is recommended for investigating vertical SNARC and SNARC-like effects (see Winter et al., 2015). The upper and lower keys were marked with the symbols * and #, respectively, so that, when participants were provided with task instructions, they were not given any explicit reference to the vertical dimension.

### Procedure

Monitor background was set to white. Each trial started with a blank screen of 800 ms, followed by a central target stimulus, which could be either a square or a diamond (width and height: 2.3°). The diamond was obtained by rotating the square 45°, to present participants with perceptually different stimuli that were otherwise identical from a physical viewpoint (see Fig. 1b). Each target stimulus remained visible for either a short (100 ms) or long (900 ms) time duration. When the target disappeared, participants responded as quickly and accurately as possible to classify the time duration of the stimulus as either short or long by pressing one of the two response keys. After a response was made or 2000 ms elapsed (whichever came first), a central visual feedback was delivered for 800 ms, consisting of a black O (width and height: 0.7°) for correct responses, a black X (width and height: 0.7°) for wrong or missed responses, or the black words “ANTICIPATED RESPONSE!” if participants responded when the target was still on the screen. Two practice blocks (12 trials each) were both followed by two experimental blocks (136 trials each), in which the stimuli were randomly selected and presented an identical number of times (2 time durations × 2 shapes × 34 repetitions). Before the first practice block, participants were presented with examples of six stimuli with short duration and six stimuli with long duration, to familiarize them with the target durations. The association between the time duration (short vs. long) and response key (bottom vs. top) was inverted in the two blocks, the order of which was counterbalanced among participants. Responses were provided using the thumbs, and the association between the thumb (left vs. right) and response key (bottom vs. top) was counterbalanced across participants and inverted in the middle of each experimental block, to avoid confounds due to manual preference. Participants were instructed to keep their thumbs placed on the buttons throughout the experiment.

### Results

The trials with anticipated responses (1% of the trials) or missed responses (0.13% of the trials) were removed. The trials with wrong responses (2.21% of the trials) were also removed and were not further analyzed due to their low percentage. The trials with correct responses and with RT 3 SD below or above the participants’ mean, computed separately for each experimental condition (1.53% of the trials), were considered to be outliers and removed.

**Fig. 2:**
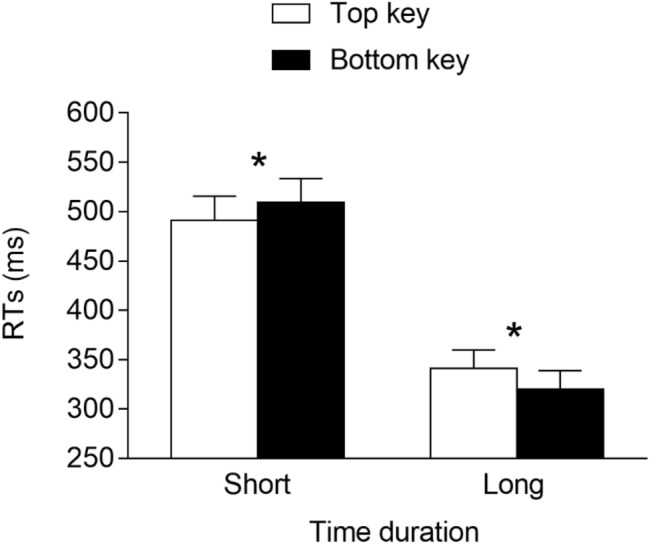
Mean RTs (error bars are SEM) observed in Experiment 1a. * = *p* < 0.05

The RTs of the correct trials were analyzed via R using linear mixed-effects models (*lme4* package; Bates et al., 2015). Here, and in the following experiments, we include the time duration (2: short vs. long), response location (2: top vs. bottom), and interaction as the fixed effects. As for the random effects, they were determined through a likelihood ratio test that compared all models ranging from the model with no random effects to the saturated model. This showed that the best model fitting data included, as the random effects, the intercept for the subjects and the by-subject slope for the time duration and response location. This model fit the data significantly better than models that did not include the by-subject slope for the time duration, *χ*^2^(3) = 946.8, *p* < 0.001, or the by-subject slope for the response location, *χ*^2^(3) = 30.3, *p* < 0.001. The analysis of this model was then carried out through an ANOVA (Type 1, Satterthwaite’s approximation for degrees of freedom; *lmerTest* package, Kuznetsova et al., 2017) devised for linear mixed-effects models. Please note that for the sake of comparison with previous studies, classic effect sizes that do not account for random effects are reported, rather than effect sizes for linear mixed-effects models. The results showed that the main effect of the time duration was significant, *F*(1, 27.0) = 99.765, *p* < 0.001, *η*^2^_*G*_ = 0.35, due to faster responses at the long (*M* = 331 ms, *SE* = 18.6) versus short (*M* = 500 ms, *SE* = 24.4) durations, whereas the main effect of the response location was not significant, *F*(1, 27) = 0.045, *p* = 0.834, *η*^2^_*G*_ ≈ 0. Importantly, the time duration × response location interaction was significant, *F*(1, 7166) = 51.650, *p* < 0.001, *η*^2^_*G*_ = 0.007, indicating the presence of a STEARC effect. The two-way interaction was further analyzed through planned comparisons (Tukey’s HSD; *lsmeans* package, Lenth, 2016) devised for linear mixed-effects models. These comparisons showed that for the short time duration, responses were faster when provided with the top key (*M* = 491 ms, *SE* = 24.7) than with the bottom key (*M* = 509 ms, *SE* = 24.3; *p* < 0.001, *d* = -−0.19), whereas the opposite result emerged for the long time duration, with faster responses associated with the bottom key (*M* = 320 ms, *SE* = 18.7) versus the top key (*M* = 341 ms, *SE* = 18.9; *p* < 0.001, *d* = −0.28; see Fig. 2).

### Discussion

Two main results emerged. First, the responses were faster overall at the long versus the short temporal duration, in line with a stimulus presentation time effect (see also, e.g., Niemi & Näätänen, 1981). More importantly, the temporal duration of the stimulus interacted with the response location, thus confirming the presence of a STEARC effect along the vertical axis. The results aligned with a top-to-bottom representation of time—specifically, the short duration was responded to more quickly with the top (vs. bottom) key, whereas the long duration was responded to more quickly with the bottom (vs. top) key. This result is consistent with the hypothesis based on reading/writing direction, rather than the hypothesis based on ATOM or the polarity correspondence model. In the next experiment, we assessed whether such a pattern could be replicated even when time was treated as an implicit dimension.

## Experiment 1b

Everything was identical to Experiment 1a, but in this case, participants were asked to classify the shape (i.e., square vs. diamond)—rather than the temporal duration (i.e., 100 vs. 900 ms)—of the target. In so doing, time became an implicit dimension, as no explicit reference to the time duration of the stimuli (i.e., short vs. long) was made in the task instructions. In this regard, it is worth recalling that previous studies suggested that both SNARC and SNARC-like effects may also emerge when the target magnitude is treated as an implicit dimension, which is interpreted as a hallmark of the automatic nature of the spatial representation of magnitudes (e.g., Dehaene et al., 1993; Fumarola et al., 2014; Sellaro et al., 2015). Please note that the implicit association between time and space has been also explored in previous studies involving the horizontal (e.g., Di Bono et al., 2012; Rolke et al., 2013) and the vertical dimensions of space (Hartmann et al., 2014a, 2014b; He et al., 2018; Stocker et al., 2016; Topić et al., 2021).

### Participants

This experiment was identical to Experiment 1a, and data collection was stopped at *N* = 30 (*Mean age* = 25 years, *SD* = 4.93, 9 males) for convenience at the end of a booking session. This was done to achieve adequate statistical power. One participant reported being left-handed, even though his EHI score (i.e., 23) indicated a slight preference for the right hand. The mean EHI of the entire sample was 66 (*SD* = 22.997, range: 23–100; please note that all EHI scores were positive). All of the participants were naïve about the purpose of the study; they had normal or correct-to-normal vision; and they provided written, informed consent approved by the local ethics committee (protocol number 1882). None of them had participated in Experiment 1a.

### Apparatus

The apparatus was the same as that used in Experiment 1a.

### Procedure

The procedure was similar to that used in Experiment 1a with only one exception: The participants were asked to classify the shape of the target stimulus (i.e., square vs. diamond) rather than its time duration.

### Results

The data were analyzed as in Experiment 1a. The trials with anticipated responses (2.71% of the trials) or missed responses (0.22% of the trials) were removed. The trials with wrong responses (1.96% of the trials) were also removed and were not further analyzed due to their low percentage. The trials with correct responses and with RT 3 SD below or above the participants’ mean, computed separately for each experimental condition (1.61% of the trials), were considered to be outliers and removed.

**Fig. 3:**
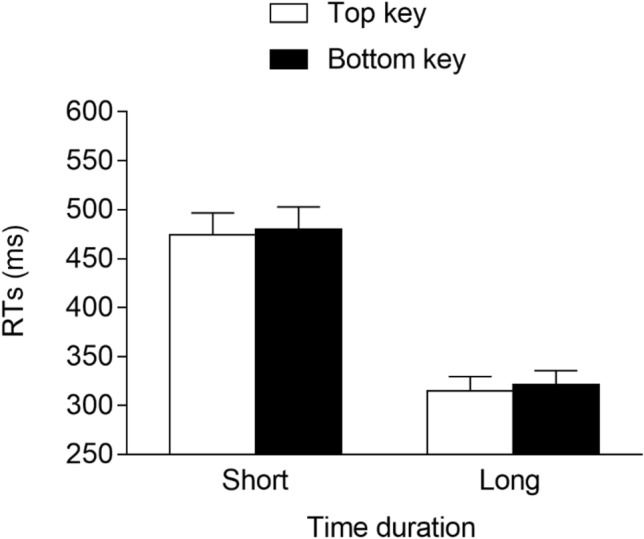
Mean RTs (error bars are SEM) observed in Experiment 1b

The best model fitting data included the time duration (2: short vs. long), response location (2: top vs. bottom), and interaction as the fixed effects. As for the random effects, they included the intercept for the subjects and the by-subject slope for the time duration. This model fit the data significantly better than a model that included, as the random effects, only the intercept for the subjects, *χ*^2^(2) = 575.0, *p* < 0.001, and it did not fit the data significantly worse than a model that included the by-subject slope for the time duration and the response location, *χ*^2^(3) = 3.3, *p* = 0.347. The main effect of the time duration was significant, *F*(1, 29.0) = 177.721, *p* < 0.001, *η*^2^_*G*_ = 0.37, due to faster responses at the long (*M* = 318 ms, *SE* = 14.4) versus the short (*M* = 477 ms, *SE* = 22.6) duration. The main effect of the response location was also significant, *F*(1, 7574.4) = 7.243, *p* = 0.007, *η*^2^_*G*_ = 0.002, due to faster responses for the top (*M* = 394 ms, *SE* = 18.1) versus the bottom (*M* = 401 ms, *SE* = 18.1) response key. Importantly, the interaction was non-significant, *F*(1, 7574.4) < 0.001, *p* = 0.997, *η*^2^_*G*_ ≈ 0, indicating the absence of a STEARC effect (see Fig. 3).

### Discussion

In this second experiment, only one result concerning time processing emerged, namely a stimulus presentation time effect (i.e., faster responses for the longer versus shorter durations). Contrary to Experiment 1a, here, the temporal duration did not interact with the response location, which indicates the absence of a vertical STEARC effect and, more broadly, that temporal duration is not automatically mapped onto the vertical space.

## Experiment 2a

Here, we wanted to provide a conceptual replication of the results that emerged in Experiment 1a but by adopting a slightly different task. A denser range of time durations was used, with four relatively short durations (i.e., 100, 200, 300, and 400 ms) and four relatively long durations (i.e., 600, 700, 800, and 900 ms). Hence, temporal durations were arranged along a continuum. Moreover, we asked the participants to determine whether the duration of the visual stimulus was either shorter or longer than a reference stimulus lasting 500 ms (i.e., a comparative judgment task). In so doing, we wanted to explore the vertical STEARC effect through a task that resembled more closely the standard approach used to reveal the SNARC effect (e.g., Dehaene et al., 1993), in which relatively small (i.e., 1, 2, 3, and 4) and large (i.e., 6, 7, 8, and 9) numbers have to be compared with the reference number of 5 (for a similar approach, based on temporal discrimination, see also Vallesi et al., 2008, Experiment 5). Considering the results that emerged in Experiment 1a, a top-to-bottom representation of time was expected to emerge in this experiment as well.

### Participants

Because we planned to collect 56 trials per condition, we needed 29 individuals to achieve adequate statistical power. We stopped at *N* = 32 (*Mean age* = 20 years, *SD* = 2.46, 8 males) for convenience at the end of a booking session to achieve adequate statistical power. One participant reported being left-handed, and the mean EHI was 63 (*SD* = 34.31, range: −67 to 100). More precisely, two participants had negative EHI scores (−4; −67), and the remaining participants had positive EHI scores (from 25 to 100). All participants were naïve about the purpose of the study; they had normal or correct-to-normal vision; and they provided written, informed consent approved by the local ethics committee (protocol number 1882). None of them had participated in Experiments 1a/b.

### Apparatus

The apparatus was the same as that used in the previous experiments.

### Procedure

**Fig. 4:**
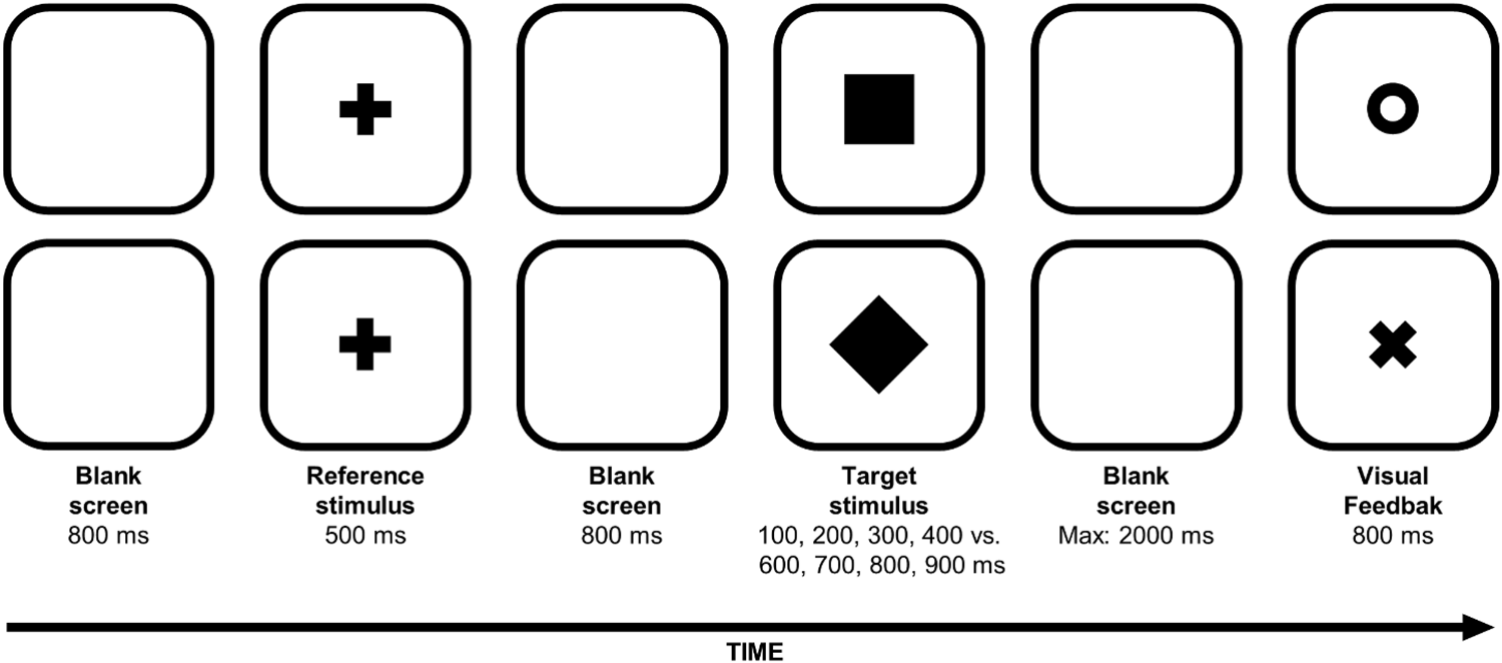
Examples of trials in which the reference stimulus (i.e., the black cross) was followed by a square shape (upper panel) and a diamond shape (lower panel). The symbols “O” and “X” were used as visual feedback for the correct versus wrong/missed responses, respectively. Stimuli not drawn to scale

The procedure was similar as that used in Experiment 1a with the following exceptions: The participants were asked to classify the duration of the target stimulus as either shorter or longer than the duration of a reference stimulus. The reference stimulus was a central black cross (width and height: 1.4°) lasting 500 ms, which was presented before the target stimulus and in between two blank screens lasting 800 ms each (see Fig. 4). Four target stimuli were shorter than the reference (i.e., 100, 200, 300, and 400 ms), and four target stimuli were longer than the reference (i.e., 600, 700, 800, and 900 ms). Each experimental block was composed of 112 trials^2^ (i.e., 8 time durations × 2 shapes × 7 repetitions).

### Results

The data were analyzed as in the previous experiments.^3^ The trials with anticipated responses (0.67% of the trials) or missed responses (0.28% of the trials) were removed. The trials with wrong responses (7.46% of the trials) were also removed and analyzed separately.^4^ The trials with correct responses and with RT 3 SD below or above the participants’ mean, computed separately for each experimental condition (1.74% of the trials), were considered to be outliers and removed.

**Fig. 5:**
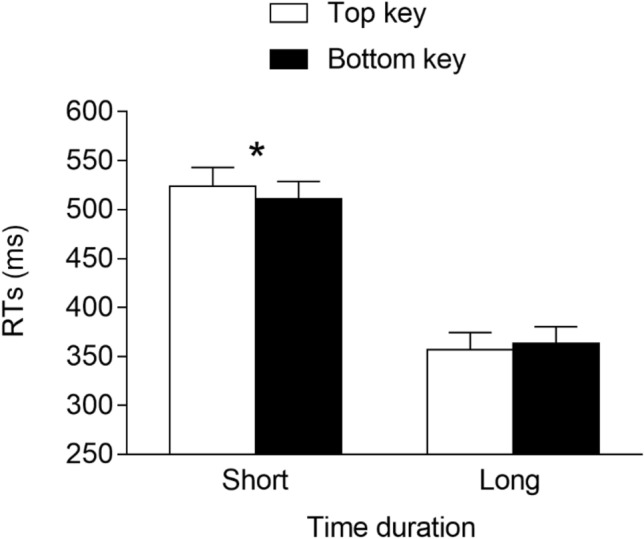
Mean RTs (error bars are SEM) observed in Experiment 2a. * = *p* < 0.05

The best model fitting data had the time duration (2: short vs. long), response location (2: top vs. bottom), and interaction as the fixed effects. As for the random effects, they included the intercept for the subjects and the by-subject slope for the time duration and response location. This model fit the data significantly better than models that did not include the by-subject slope for the time duration, *χ*^2^(3) = 172.2, *p* < 0.001, or the by-subject slope for the response location, *χ*^2^(3) = 30.96, *p* < 0.001. The main effect of the time duration was significant, *F*(1, 30.9) = 280.052, *p* < 0.001, *η*^*2*^_*G*_ = 0.80, due to faster responses at the long (*M* = 360 ms, *SE* = 17.0) versus short (*M* = 517 ms, *SE* = 18.1) durations, whereas the main effect of the response location was not significant, *F*(1, 30.7) = 0.536, *p* = 0.47, *η*^2^_*G*_ = 0.001. Importantly, the time duration × response location interaction was significant, *F*(1, 6357.8) = 8.942, *p* = 0.003, *η*^2^_*G*_ = 0.016, indicating the presence of a STEARC effect. Planned comparisons showed that for the short time durations, the responses were faster when provided with the bottom key (*M* = 511 ms, *SE* = 17.7) versus the top key (*M* = 524 ms, *SE* = 19.0; *p* = 0.03, *d* = -−0.17), whereas for the long time durations, no significant differences emerged between the responses provided with the bottom key (*M* = 363 ms, *SE* = 17.3) and the top key (*M* = 357 ms, *SE* = 17.3; *p* = 0.384, *d* = 0.08; see Fig. 5),^5^.^6^

### Discussion

The presence of a stimulus presentation time effect was confirmed, and more importantly, the temporal duration interacted with the response location, thus revealing the presence of a vertical STEARC effect. However, and contrary to what was observed in Experiment 1a, the representation of time here followed a bottom-to-top direction. The shorter temporal durations were responded to more quickly with the bottom than with the top key, whilst, for the longer duration, no differences between the two response keys emerged. The results of Experiment 2a align with the hypothesis based on ATOM and the polarity correspondence model, and with the general principle that “more is up”. To identify what may have caused the reversal of the STEARC effect reported here, we reasoned that a substantial difference between Experiments 1a and 2a is that in the latter one, the task required participants to compare the temporal duration of the target with that of the reference. Hence, we felt that the potential role of the reference in shaping results needed to be properly addressed. However, similar to Experiment 1b, we also deemed it important to establish, through the next experiment, whether the same pattern of results reported here could emerge even when the temporal dimension was treated implicitly rather than explicitly.

## Experiment 2b

Everything was identical to Experiment 2a with the only exception that the target stimuli were classified on the basis of shape rather than temporal duration (i.e., as in Experiment 1b).

### Participants

This experiment was identical to Experiment 2a, and data collection was therefore stopped at *N* = 30 (*Mean age* = 23 years, *SD* = 7.46, 8 males) for convenience at the end of a booking session to achieve adequate statistical power. Three participants reported being left-handed, and the mean EHI was 52 (*SD* = 49.44, range: −100 to 100). More precisely, three participants had negative EHI scores (−47; −76; −100), and the remaining participants had positive EHI scores (from 4 to 100). All participants were naïve about the purpose of the study; they had normal or correct-to-normal vision; and they provided written, informed consent approved by the local ethics committee (protocol number 1882). None of them had participated in the previous experiments.

### Apparatus

The apparatus was the same as that used in the previous experiments.

### Procedure

The procedure was identical to that used in Experiment 2a with only one exception: The participants were asked to classify the shape of the target stimulus (i.e., square vs. diamond) instead of its time duration (see also Experiment 1b).

### Results

The data were analyzed as in the previous experiments. The trials with anticipated responses (3.39% of the trials) or missed responses (0.24% of the trials) were removed. The trials with wrong responses (1.41% of the trials) were also removed and were not further analyzed due to their low percentage. The trials with correct responses and with RT 3 SD below or above the participants’ mean, computed separately for each experimental condition (1.54% of the trials), were considered to be outliers and removed.

**Fig. 6:**
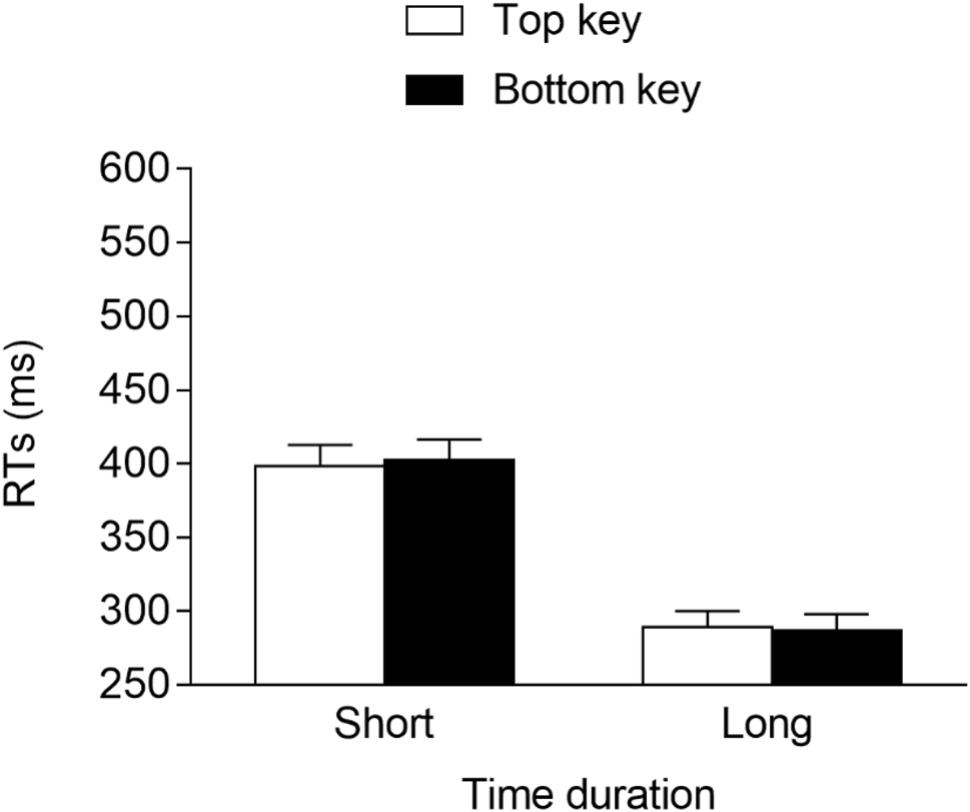
Mean RTs (error bars are SEM) observed in Experiment 2b

The best model fitting data had the time duration (2: short vs. long), response location (2: top vs. bottom), and interaction as the fixed effects. As for the random effects, they included the intercept for the subjects and the by-subject slope for the time duration. This model fit the data significantly better than a model that included, as the random effects, only the intercept for the subjects, *χ*^2^(2) = 248.0, *p* < 0.001, and it did not fit the data significantly worse than a model that included the by-subject slope for the time duration and the response location, *χ*^2^(3) = 6.9, *p* = 0.074. The main effect of the time duration was significant, *F*(1, 29.0) = 135.220, *p* < 0.001, *η*^2^_*G*_ = 0.44, due to faster responses at the long (*M* = 288 ms, *SE* = 10.8) versus the short (*M* = 400 ms, *SE* = 14.3) durations. Neither the main effect of the response location, *F*(1, 6221.5) = 0.111, *p* = 0.739, *η*^2^_*G*_ ≈ 0, nor the interaction, *F*(1, 6221.5) = 0.913, *p* = 0.339, *η*^2^_*G*_ = 0.002, was significant (see Fig. 6), thus excluding the presence of a STEARC effect.

### Discussion

The results mimicked what emerged in Experiment 1b. A stimulus presentation time effect clearly occurred while the temporal duration did not interact with the response location, thus indicating the absence of a vertical STEARC effect. This appears to confirm that time durations are not mapped onto a vertical space in an automatic fashion. For this reason, in all of the subsequent experiments, the task required explicitly classifying the temporal duration of the target, even if the two shapes (i.e., square and diamond) were kept for consistency with the experiments conducted so far.

## Experiment 3

This experiment was specifically designed to explore whether the presence of a temporal reference, to be used to classify the temporal duration of the target stimulus, may have played a role in shaping the bottom-to-top STEARC effect reported in Experiment 2a. In this experiment, everything was identical to Experiment 1a except that a visual temporal reference of 500 ms (i.e., the black cross employed in Experiments 2a/b) was also provided at the beginning of each trial. The participants received the specific instruction to judge whether the target stimulus lasted for a shorter (i.e., 100 ms) or a longer (i.e., 900 ms) temporal duration with respect to the reference. If the bottom-to-top time representation reported in Experiment 2a was due to the presence of the reference, then evidence of a similar representation of time should also emerge in Experiment 3.

### Participants

Because the number of the trials in this experiment was identical to that in Experiments 2a/b, data collection was stopped at *N* = 30 (*Mean age* = 21 years, *SD* = 1.92, 9 males) for convenience at the end of a booking session to achieve adequate statistical power. Two participants reported being left-handed, and the mean EHI was 62 (*SD* = 38.6, range: −100 to 100). More precisely, two participants had negative EHI scores (−25; −100), and the remaining participants had positive EHI scores (from 20 to 100). All of the participants were naïve about the purpose of the study; they had normal or correct-to-normal vision; and they provided written, informed consent approved by the local ethics committee (protocol number 1882). None of them had participated in the previous experiments.

### Apparatus

The apparatus was the same as that used in the previous experiments.

### Procedure

The procedure was similar to that used in Experiment 1a with the following exception: A visual temporal reference of 500 ms (i.e., the same black cross employed in Experiments 2a/b) appeared at the beginning of each trial, and the participants were explicitly instructed to compare the temporal duration of the target stimulus with that of the reference.

### Results

The data were analyzed as in the previous experiments. The trials with anticipated responses (0.79% of the trials) or missed responses (0.13% of the trials) were removed. The trials with wrong responses (1.48% of the trials) were also removed and were not further analyzed due to their low percentage. The trials with correct responses and with RT 3 SD below or above the participants’ mean, computed separately for each experimental condition (1.93% of the trials), were considered to be outliers and removed.

**Fig. 7:**
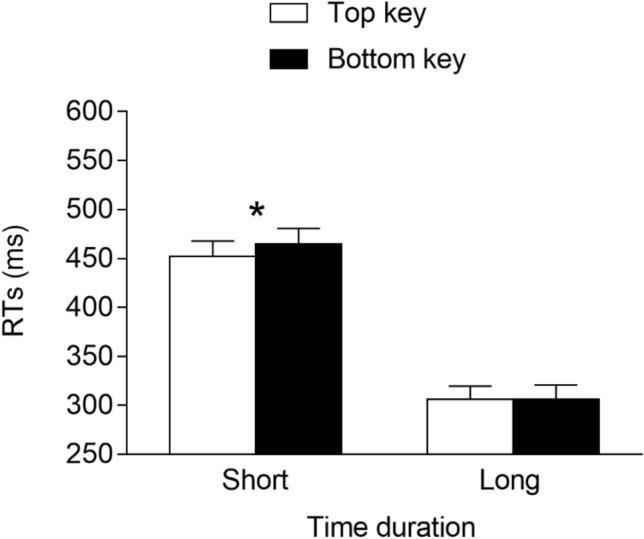
M*e*an RTs (error bars are SEM) observed in Experiment 3

The best model fitting data included the time duration (2: short vs. long), response location (2: top vs. bottom), and interaction as the fixed effects. As for the random effects, they included the intercept for the subjects and the by-subject slope for the time duration and response location. This model fit the data significantly better than models that did not include the by-subject slope for the time duration, *χ*^2^(3) = 297.4, *p* < 0.001, or the by-subject slope for the response location, *χ*^2^(3) = 34.3, *p* < 0.001. The main effect of the time duration was significant, *F*(1, 29.0) = 243.912, *p* < 0.001, *η*^*2*^_*G*_ = 0.44, due to faster responses for the long (*M* = 306 ms, *SE* = 13.9) versus the short (*M* = 458 ms, *SE* = 15.5) duration, whereas the main effect of the response location was not significant, *F*(1, 28.9) = 2.159, *p* = 0.153, *η*^2^_*G*_ = 0.002. Importantly, the time duration × response location interaction was significant, *F*(1, 7031.0) = 7.778, *p* = 0.005, *η*^2^_*G*_ = 0.002, indicating the presence of a STEARC effect. Planned comparisons showed that for the short time duration, the responses were faster when provided with the top key (*M* = 452 ms, *SE* = 15.8) versus the bottom key (*M* = 465 ms, *SE* = 15.7; *p* = 0.008, *d* = -−0.20), whereas for the long time duration, no differences emerged for the responses associated with the bottom key (*M* = 306 ms, *SE* = 14.7) and the top key (*M* = 306 ms, *SE* = 13.5; *p* = 0.912, *d* ≈ 0; see Fig. 7).

### Discussion

This experiment provided a conceptual replication of the pattern reported in Experiment 1a. Besides the presence of a stimulus presentation time effect, the temporal duration interacted with the response location, and the direction of the vertical STEARC indicated a top-to-bottom spatial representation of time. Hence, at first sight, it could be inferred that the adoption of the reference stimulus did not play any major role in shaping the direction of the spatial representation of time, although the strength of the top-to-bottom STEARC effect appeared to be slightly reduced with respect to Experiment 1a. Nevertheless, here, the temporal durations of the two targets could easily be discriminated against each other. Therefore, though participants were explicitly instructed to compare the duration of the target with that of the reference, one could argue that the task could be successfully completed without necessarily taking the reference into account. If this hypothesis is correct, then this would mean that the mechanisms shaping the participants’ responses were more similar to those in Experiment 1a than in Experiment 2a, which, in turn, would explain why the results observed here mirror those that emerged in our first experiment. The next experiment was devised with the aim of further strengthening the relevance of the temporal reference for the task at hand.

## Experiment 4

Everything was identical to Experiment 3, but the two temporal durations that were closest—and more similar—to the reference in Experiment 2a/b (i.e., 400 and 600 ms) were also employed with the aim of creating a context in which participants were strongly incentivized to rely on the reference to complete the task successfully. A bottom-to-top representation of time was expected, in line with Experiment 2a.

### Participants

Because the number of trials in this experiment was identical to that in Experiments 2a/b and 3, data collection was stopped at *N* = 34 (*Mean age* = 21 years, *SD* = 1.8, 13 males) at the end of a booking session to achieve adequate statistical power. One participant reported being left-handed, and the mean EHI was 66 (*SD* = 28.74, range: −43 to 100). More precisely, one participant had a negative EHI score (−43), and the remaining participants had positive EHI scores (from 26 to 100). All of the participants were naïve about the purpose of the study; they had normal or correct-to-normal vision; and provided written, informed consent approved by the local ethics committee (protocol number 1882). None of them had participated in the previous experiments.

### Apparatus

The apparatus was the same as that used in the previous experiments.

### Procedure

The procedure was identical to that used in Experiment 3 with the following exception: Two additional temporal durations (i.e., 400 vs. 600 ms) were also used.

### Results

The data were analyzed as in the previous experiments. The trials with anticipated responses (0.66% of the trials) or missed responses (0.17% of the trials) were removed. The trials with wrong responses (13.27% of the trials) were also removed and analyzed separately.^7^ The trials with correct responses and with RT 3 SD below or above the participants’ mean, computed separately for each experimental condition (1.93% of the trials), were considered to be outliers and removed.

**Fig. 8:**
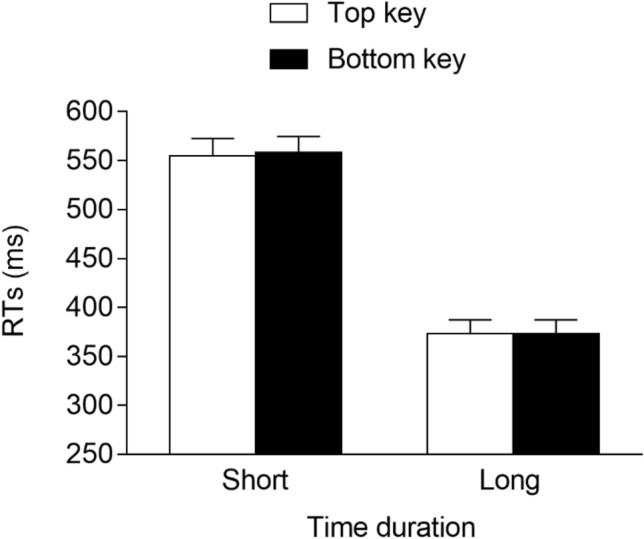
Mean RTs (error bars are SEM) observed in Experiment 4

The best model fitting data included the time duration (2: short vs. long), response location (2: top vs. bottom), and interaction as the fixed effects. As for the random effects, they included the intercept for the subjects and the by-subject slope for the time duration and response location. This model fit the data significantly better than models that did not include the by-subject slope for the time duration, *χ*^2^(3) = 121.3, *p* < 0.001, or the by-subject slope for the response location, *χ*^2^(3) = 44.9, *p* < 0.001. The main effect of the time duration was significant, *F*(1, 33.0) = 367.823, *p* < 0.001, *η*^2^_*G*_ = 0.44, due to faster responses at the long (*M* = 373 ms, *SE* = 13.6) versus the short (*M* = 556 ms, *SE* = 16.5) time duration. Neither the main effect of the response location, *F*(1, 33.2) = 0.050, *p* = 0.824,, *η*^2^_*G*_ = 0.002, nor the interaction, *F*(1, 6326.5) = 0.220, *p* = 0.64, *η*^2^_*G*_ = 0.002, was significant (see Fig. 8).

Additional analyses were also performed by considering the two farthest (i.e., 100 vs. 900 ms) and closest (i.e., 400 vs. 600 ms) temporal durations from the reference separately to assess whether a STEARC effect was detectable in at least one of the two conditions. In both cases, the best model fitting data included the time duration (2: short vs. long), response location (2: top vs. bottom), and interaction as the fixed effects. As for the random effects, they included the intercept for the subjects and the by-subject slope for the time duration and response location. These models fit the data significantly better than models that did not include the by-subject slope for the time duration or for the response location, *χ*^2^s > 28.4, *p*s < 0.001. Furthermore, in both cases, the main effect of the time duration was significant (*p*s < 0.001), whereas the main effect of the response location (*p*s > 0.562) and the interaction term (*p*s > 0.659) were both not significant. This latter result confirmed the overall absence of the STEARC effect.

### Discussion

The results confirmed the presence of a stimulus presentation time effect. However, and surprisingly, the temporal duration and response side did not interact, and therefore, no evidence of a vertical STEARC effect emerged. The absence of a STEARC effect was confirmed not only for the two intermediate temporal durations (i.e., 400 and 600 ms) but also for the two more extreme temporal durations (i.e., 100 and 900 ms), for which a reliable STEARC effect emerged in three of the previous experiments (i.e., Experiments 1a, 2a, and 3). Overall, it appears that rather than the reference, other features of the task would be involved in determining the emergence (if any) of the STEARC effect and its eventual spatial direction.

As for the present context, it is important to note that the set of temporal durations used here represents a peculiar subset of the temporal stimuli employed in Experiment 2a, as only the two closest (i.e., 400 and 600 ms) and farthest (i.e., 100 and 900 ms) temporal durations, relative to the 500-ms midpoint, were employed. With that, one could suggest that participants were immersed in a context in which the different temporal durations could not easily be interpreted as belonging to either a clear and defined dichotomy—such as in Experiment 1a, in which a top-to-bottom STEARC effect emerged—or a denser set of stimuli arranged along a hypothetical continuum—such as in Experiment 2a, in which a bottom-to-top STEARC effect emerged instead. Hence, we tentatively suggest that such an ambiguous context may have somehow interfered with building up a clear and defined mental representation of time.

Taken together, the results emerging from this experiment and the previous ones of the present work outline a rather peculiar vertical representation of time, which suggests that mental representations of time may vary depending on the context. A possible feature that seems to shape the vertical STEARC effect could be related to how the target temporal durations would be arranged within a given array—for example, two antipodes or several stimuli in succession. Despite this intriguing perspective, which is further discussed in the general discussion section, we felt the need to conduct some final experiments that confirmed that the experimental paradigm we devised was also suitable for revealing the well-documented left-to-right STEARC effect along the horizontal axis (i.e., Experiments 5–7; for the sake of parsimony, these three experiments are reported in the Appendix). A left-to-right STEARC effect emerged when temporal durations were 1 s vs. 3 s (Experiment 5; see also Vallesi et al., 2008, for same time durations), 100 ms vs. 900 ms (Experiment 6), and when the 100–400 ms vs. 600–900 ms ranges were used (Experiment 7).

## General discussion

Time is a crucial dimension that deeply shapes the human mind and human behavior (e.g., Grondin, 2010). Although time is, by definition, an abstract construct, several works have shown that it can be mapped onto space. Most of these works focused on the horizontal axis, reporting that time concepts, such as *before* or *short*, can be represented on the left side of the space, whereas concepts such as *after* or *long* can be represented on the right side (e.g., Bender & Beller, 2014; Bonato et al., 2012; Núñez & Cooperrider, 2013). Fewer works focused on the vertical dimension. They led, for instance, to non-conclusive results when the task required the categorization of temporal durations (e.g., Ishihara et al., 2008), or reported evidence of the vertical spatial representation of time confined to individuals who possess specific metaphors that couple time with vertical locations. An example of this can be found in the case of Mandarin speakers for whom, e.g., “early is up” and “later is down” (Boroditsky et al., 2011; Sun & Zhang, 2021).

The current work represents an attempt to unveil, through a systematic and comprehensive approach, a vertical spatial representation of time by adopting a novel task based on the discrimination of time durations. In six experiments, a central target visual stimulus (i.e., square vs. diamond) could last for a variable temporal duration. Participants were required to classify either the actual length of the temporal duration associated with the target (i.e., time was treated as an explicit dimension; Experiments 1a, 2a, 3, and 4) or the shape of the target (i.e., time was treated as an implicit dimension; Experiments 1b and 2b). Manual responses were collected through a response box with the keys genuinely placed along the vertical axis, thus avoiding the possible confound with the sagittal axis that was present in previous work (see Beracci et al., 2021a, 2021b; Ishihara et al., 2008). Moreover, stimuli were always presented at the center of the screen, thus avoiding the possibility that vertical STEARC effects could be related to the vertical arrangement of the stimuli (see Beracci et al., 2021a, 2021b; Casasanto & Bottini, 2014). In addition, to confirm that our task was able to elicit the well-known left-to-right STEARC effect, three further experiments (Experiments 5–7; see the Appendix) were carried out in which manual responses were collected through horizontally placed response keys. In the following paragraphs, the main results emerging from this set of experiments are discussed. For the sake of clarity, the discussion deals with the more general results first and then approaches the peculiar patterns of the results concerning the vertical STEARC effect.

First, a robust and consistent effect emerging in all of the experiments was a stimulus presentation time effect (see also, e.g., Niemi & Näätänen, 1981). Participants were overall faster in responding to targets associated with longer versus shorter time durations. This is a well-known and classic effect, basically characterizing all reaction time tasks in which participants are allowed—with a relatively variable temporal duration—between a so-called warning signal (in the present context represented by the onset of the target stimulus) and an imperative signal associated with the response (in the present context represented by the offset of the target stimulus). Within the variable temporal duration, participants are assumed to prepare for the response on the basis of a temporal expectation centered on the time that elapses after the onset of the warning stimulus (see also, e.g., Niemi & Näätänen, 1981; Vallesi et al., 2007). This would explain the faster responses that emerge at longer time durations, for which participants are assumed to be able to make more precise time expectations.

Second, when the temporal duration of the target was treated as an implicit dimension (i.e., the participants classified the shape rather than the duration of the target; Experiments 1b and 2b), no evidence of a STEARC effect emerged. This contrasts with the relatively vast literature showing that both SNARC and SNARC-like effects can still be detected even when the participants’ responses are not based on the actual magnitude associated with a given dimension (e.g., number or physical size) but rather on another feature of the stimuli (e.g., even vs. odd number, animate vs. inanimate stimulus, target position; see, e.g., Dehaene et al., 1993; Sellaro et al., 2015; Topić et al., 2021). It is important to note that in both Experiments 1b and 2b, time—despite being an implicit dimension—was still a task-relevant variable because to successfully complete the task, participants had to wait until the visual stimulus disappeared from the screen to provide their responses. Nevertheless, it seems that the mere request to classify the stimulus on the basis of its shape, rather than on the temporal duration, was enough to prevent the vertical spatial representation of time to emerge. This would suggest that the spatial representation of time is not automatic and mandatory. Rather, it is something that occurs as a consequence of the explicit processing of time-relevant information.

Third, when the temporal duration of the target was treated as an explicit dimension and the participants provided a response along the *horizontal* axis, supporting evidence of a horizontal STEARC effect emerged (Experiments 5–7; see the Appendix). In all three of these experiments, which were characterized by different sets of temporal stimuli, the time duration interacted with the response side, indicating the presence of a left-to-right spatial representation of time. The results obtained in these three experiments are of great relevance because they confirm that the paradigm devised—based on peculiar stimuli and time durations—is suitable for eliciting a horizontal STEARC effect that is fully consistent with that reported in previous studies (e.g., Santiago et al., 2007; Vallesi et al., 2008; Weger & Pratt, 2008; Zhao et al., 2018). In particular, we highlight that similar results emerged both when the temporal durations were the same as those adopted in Vallesi et al.’s (2008) Experiment 1 (i.e., 1 vs. 3 s) and when the temporal durations belonged to the 100–900 ms temporal range that was adopted in the present work. Hence, we are confident that the overall and peculiar pattern of results stemming from our experiments on the vertical STEARC effect was actually shaped by the relationships between the relative time durations of the target stimuli, rather than by other trivial—and somewhat arbitrary—features of the stimuli, such as their visual appearance (i.e., shape, size, color) or absolute durations.

Fourth—and of particular interest for our main goal—when the temporal duration of the target was treated as an explicit dimension and the participants provided a response along the *vertical* axis, supporting evidence of a vertical STEARC effect emerged (Experiments 1a, 2a, 3). However, such an effect did not unfold following a single and unambiguous spatial direction—such as in the case of the consistent left-to-right direction emerging in Experiments 5–7 (see the Appendix). Rather, two opposite spatial representations of time occurred. In Experiment 1a, in which only one short (100 ms) and one long (900 ms) time duration were employed, a top-to-bottom spatial representation of time arose, which was consistent with the canonical reading direction along the vertical axis (i.e., from top to bottom). On the contrary, the results provided in Experiment 2a, which employed both shorter (100–400 ms) and longer (600–900 ms) time durations compared with a 500-ms time reference, speak in favor of an opposite bottom-to-top spatial representation. Initially, we identified the request to compare the duration of the target with that of a reference as the possible cause of such an inversion. Despite this, in the subsequent Experiment 3—which was identical to Experiment 1a with the additional request of completing the classification task while relying on the 500-ms time reference—a top-to-bottom spatial representation of time still emerged. Moreover, no evidence of a spatial representation of time emerged in Experiment 4, which was identical to Experiment 3 with the addition of the 400 and 600 ms target durations. Hence, we feel that the potential role of the temporal reference in shaping the direction of the spatial representation of time along the vertical axis should be dismissed. As mentioned in the discussion section of Experiment 4, besides the absence versus presence of a time reference, another relevant difference between Experiments 1a and 2a is that in the former case, the two target durations were clearly different from each other, thus creating a defined dichotomy, whereas in the latter case, the denser set of target durations, composed of eight values (plus the reference), may have induced the perception of a temporal continuum. Consequently, perceiving the temporal durations as a dichotomy versus a continuum could be the key element for explaining the activation of one spatial representation of time over the other. In the next subsection, we will outline in more detail a reasoning aimed at supporting this interpretative key.

### Dichotomies versus continua: can stimuli distribution shape space–time associations?

We start by noting that indirect support of the possible dichotomy versus continuum explanation could be found in two recent studies, both involving Mandarin speakers, which we were not aware of when we planned our own work (i.e., Ding et al., 2020; Xiao et al., 2018).

Xiao et al. (2018) observed an inversion of the spatial representation of time—similar to that reported here—across two experiments. In a first experiment, participants were asked to classify—by pressing one of two vertically aligned response keys—a central picture (e.g., a half-eaten apple) as either occurring earlier or later than a previously seen picture (e.g., a whole apple). A top-to-bottom temporal representation emerged, with the top part of the space associated with *earlier* and the bottom with *later*. However, in a second experiment, participants (all young adults) were asked to classify nine episodes of their lives as either belonging to the past, such as elementary school (i.e., an early event along the mental timeline), or to the future, such as middle age (i.e., a later event along the mental timeline). The representation of time went from bottom to top because past (the early time) events were associated with the bottom part of the space, and future (the later time) events were associated with the top part. Despite this novel evidence, it is important to note that the primary goal of Xiao et al. (2018) was the exploration of the spatial representation of time along the sagittal axis, and therefore, the inversion along the vertical was only reported anecdotally.

A past-bottom/future-top mapping was also reported by Ding et al. (2020) in three experiments employing 10 past-related words and 10 future-related words (see also Beracci & Fabbri, 2022, for similar results with Italian participants). When discussing the results of their experiments, Ding et al. (2020) concluded that *time-referenced-point* tasks, such as those involving the pictures of actions or events forming a definite temporal sequence (e.g., the picture of a whole apple, followed by the picture of a half-eaten apple), would give rise to a top-to-bottom mapping, whereas *ego-referenced-point* tasks, such as those involving abstract concepts referring to participants’ past versus future events, would give rise to a bottom-to-top mapping.

Although the reasoning pushed forward by Ding et al. (2020) can be applied not only to their study but also to Xiao et al. (2018), the results of our experiments seem to require a broader principle explaining how time classification tasks might actually shape the direction of the vertical STEARC effect. Indeed, the different vertical STEARC effects observed here, emerged despite having employed relatively simple visual stimuli whose time durations cannot easily be represented as sequences of events (e.g., before vs. after, as in *time-referenced-point* tasks) or as participants’ past versus future events (as in *ego-referenced-point* tasks). For these reasons, the more general explanation based on perceiving temporal stimuli as belonging to either a dichotomy or a continuum could be more suitable for justifying both ours and previous results (Ding et al., 2020; Xiao et al., 2018).

To recap, top-to-bottom time representations—which are consistent with the reading direction—would emerge for stimuli that can be embedded within a defined temporal dichotomy, such as in the case of the 100 versus 900 ms time durations used in our Experiments 1a and 3, which probably give rise to a short versus long dichotomy, or in the case of the pictures showing the earlier versus later stages of actions or events used in Xiao et al. (2018; Experiment 1), which probably give rise to an earlier versus later dichotomy. By contrast, bottom-to-top representations—which are consistent with both ATOM and the polarity correspondence model, and with the general “more is up” metaphor—would emerge for stimuli that can evoke a relatively dense temporal continuum, like the nine time durations used in Experiment 2a, the nine life episodes used in Xiao et al. (2018; Experiment 2), or the 20 time-related words used in Ding et al. (2020) and in Beracci and Fabbri (2022). In this regard, the results of Experiment 4—in which no evidence of a spatial representation of time emerged, likely because the temporal context that the employed target durations created was rather ambiguous—are of particular interest, and they seem to corroborate the intriguing perspective here outlined (i.e., in Experiment 4, the participants may have perceived the temporal durations as belonging to neither a dichotomy nor a continuum).

Notably, none of the theories of space–magnitude associations considered here (i.e., ATOM, polarity correspondence, “reading/writing direction”) can account for the results emerging from the present set of experiments. In fact, these theories would predict either a bottom-to-top representation (ATOM and polarity correspondence) or a top-to-bottom representation (“reading/writing direction”), independently of whether the stimuli are perceived as belonging to a dichotomy or to a continuum. A tentative theoretical interpretation of why this may affect the direction of the vertical STEARC effect is provided in the next subsection. That said, to the best of our knowledge, the studies by Ding et al. (2020) and Xiao et al. (2018), together with what is reported here, are currently the only works providing evidence for a malleable representation of time along the vertical axis. Therefore, any solid conclusion should be reported with caution, and future studies are necessary to provide additional support for this novel phenomenon.

### Magnitude continua and the “more is up” metaphor

Assuming that our hypothesis is correct, a major goal for future studies will be to reveal the specific reasons *why* different representations of time-related stimuli (i.e., dichotomy vs. continuum) should lead to opposite spatial representations along the vertical axis. In this regard, we speculate that this might be related to how everyday life experiences shape the “more is up” concept. It has been suggested that we would learn that “more is up” through specific real-life experiences, such as stacking objects on top of each other, or pouring a liquid into a glass (see, e.g., Myachykov, 2014). All of these experiences appear to be intrinsically related to the notion of magnitudes that gradually increase from the bottom part of the space to the top one. Hence, it can be hypothesized that “more is up” is spontaneously activated only by stimuli that can be arranged along a continuum.

By contrast, stimuli that more logically belong to a dichotomy would be less than ideal for activating the “more is up” concept. When dichotomous stimuli are used, the most salient direction of the vertical space would coincide with a top-to-bottom spatial vector, which ideally recalls the reading direction. In other words, when dichotomous stimuli are used, the most salient vertical direction of space would be top-to-bottom because this coincides with the direction related to a well-learned and highly practiced activity, such as reading. However, when stimuli that can be hypothetically arranged along a continuum are used, the saliency of this vector would be overtaken by the saliency of the bottom-to-top vector, as bottom-to-top is the direction typically associated with experiences with gradually increasing magnitudes. This hypothesis is also consistent with the idea that mapping time onto the most salient dimension of space would enhance the global coherence of the space–time representation, thus facilitating the encoding of the stimuli (see also Torralbo et al., 2006). Furthermore, the hypothesis is also consistent with the idea that the way a source domain (e.g., space) and target domain (e.g., time) are mentally represented is determined by the way the two domains are correlated in experience (the correlation in experience principle, or CORE; Pitt & Casasanto, 2020).

### Unbalanced patterns of results in SNARC-like tasks

As a conclusive note, we report that although in some cases the comparisons between the “top versus bottom” or “left versus right” keys were significant for both short and long durations (see Experiments 1a and 7; see also Vallesi et al., 2008), in other cases they were significant only for short durations. This emerged for vertical as well as horizontal responses (see Experiments 2a, 3, 5, and 6).

The possibility of reporting unbalanced response patterns when SNARC-like tasks are adopted is not so rare (e.g., Chang & Cho, 2015; Dalmaso et al., 2022; Dalmaso & Vicovaro, 2019; Giuliani et al., 2021; Ren et al., 2011). Recently, Giuliani et al. (2021) speculated that this may be due to the fact that processing nonnumerical dimensions would be less direct and unambiguous compared with processing numbers. This, in turn, could be reflected in relatively weak associations connecting some dimensions with space. Another possible explanation, which can be applied to our context, considers the fact that, at longer durations, it was possible to prepare a response before the stimulus had finished being presented (but the participants had to refrain from providing the response). This aspect of our experimental design, which was missing at shorter durations, may have made the STEARC effect disappear. In any case, to the best of our knowledge, so far, no studies have been conducted with the aim of providing a systematic explanation for such unbalanced patterns, thus leaving room for numerous interpretations.

Nevertheless, it is worth mentioning that the stronger association with space for stimuli associated with relatively slow response latencies (i.e., the shorter temporal durations in the present context) than for stimuli associated with relatively fast latencies (i.e., the longer stimuli employed in the present context) is in line with previous studies showing that the SNARC effect is typically stronger for stimuli associated with relatively slow—rather than fast—response latencies (e.g., Gevers et al., 2006). This indicates that SNARC and STEARC effects are similar in this regard. Moreover, differences in performance that generally emerge when processing relatively smaller and larger quantities (i.e., the size effect; Moyer & Landauer, 1967) may have also played a role.

## Conclusion

We have reported novel evidence of a STEARC effect along the vertical axis when Western participants were asked to classify the lengths of different temporal durations. Intriguingly, the direction of this effect varied across the experiments, as both a top-to-bottom and a bottom-to-top spatial representation of time emerged, likely due to the different natures (dichotomy vs. continuum) of the employed temporal stimuli. Hence, the vertical STEARC effect appears to be a malleable rather than a rigid and unmodifiable instance.

## Figures and Tables

**Fig. 9:**
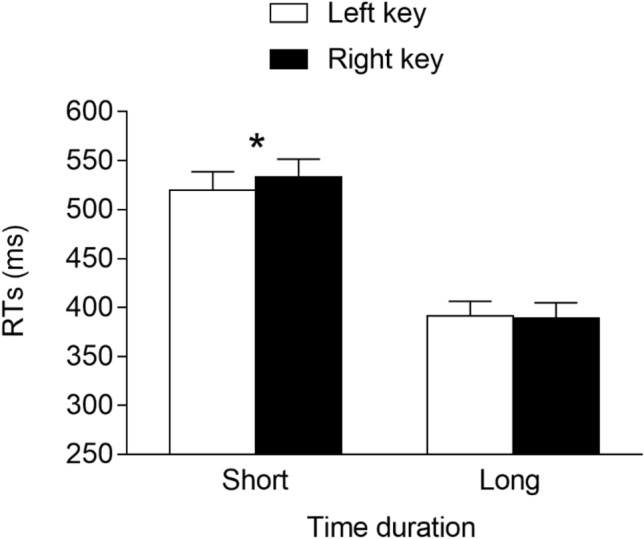
Mean RTs (error bars are SEM) observed in Experiment 5. * = *p* < 0.05

**Fig. 10:**
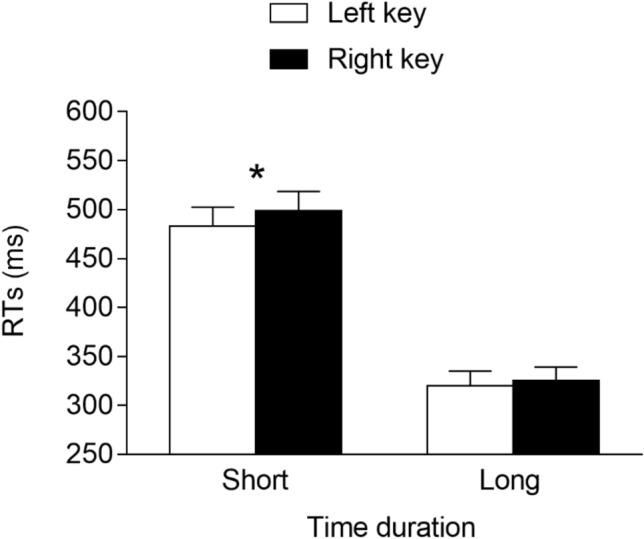
Mean RTs (error bars are SEM) observed in Experiment 6. * = *p* < 0.05

**Fig. 11:**
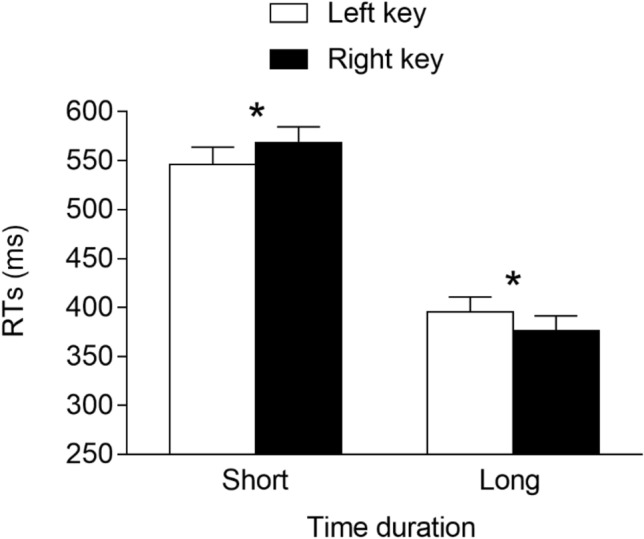
Mean RTs (error bars are SEM) observed in Experiment 7. *p < 0.05

## Data Availability

This study was not preregistered. The data associated with this work can be found on OSF at the following link: https://www.doi.org/10.17605/OSF.IO/KM2BG

## Appendix

In this Appendix, we describe three additional experiments (Experiments 5–7) on the STEARC effect along the horizontal axis.

## Experiment 5

The main aim of this experiment was to provide a conceptual replication of the Experiment 1 appearing in Vallesi et al. (2008), which mainly inspired our work. Unlike our Experiments 1a–4, in the first experiment of Vallesi et al. (2008), horizontal manual responses and relatively long temporal durations (i.e., 1 vs. 3 s) were employed. Here, everything was identical to Experiment 1a with the following two exceptions: Manual responses were provided along the horizontal axis, and the target temporal duration could be either one second or three seconds.

### Participants

Because the number of the trials in this experiment was identical to that of Experiments 1a/b, data collection was stopped at *N* = 32 (*Mean age* = 25 years, *SD* = 1.1, 16 males) for convenience to achieve adequate statistical power. One participant reported being left-handed, and the mean EHI was 80 (*SD* = 25.17, range: −25 to 100). More precisely, one participant had a negative EHI score (−25), and the remaining participants had positive EHI scores (from 50 to 100). All participants were naïve about the purpose of the study; they had normal correct-to-normal vision; and they provided written, informed consent approved by the local ethics committee (protocol number 1882). None of them had participated in the previous experiments.

### Apparatus

The experiment was programmed with PsychoPy and delivered online through Pavlovia, which are known to provide highly precise and reliable behavioral data (Bridges et al., 2020).

### Procedure

Everything was identical to Experiment 1a, with three exceptions. First, the short and long durations were one second and three seconds, respectively, and second, manual responses were provided along the horizontal axis using the standard keyboard of the PC (i.e., as in Vallesi et al., 2008). In particular, the response keys were “H” and “K”—two buttons whose distance was similar to that of the two response buttons used in the vertical response box employed in the previous experiments. Third, because the experiment was delivered online, the short version of the EHI (Veale, 2014) was adopted, which offers good reliability and the advantage of providing participants with both instruction and response options in simplified versions.

### Results

The data were analyzed as in the previous experiments. The trials with anticipated responses (0.32% of the trials) or missed responses (0.16% of the trials) were removed. The trials with wrong responses (3.79% of the trials) were also removed and were not further analyzed due to their low percentage. The trials with correct responses and with RT 3 SD below or above the participants’ mean, computed separately for each experimental condition (1.65% of the trials), were considered to be outliers and removed.

The best model fitting data included the time duration (2: short vs. long), response side (2: left vs. right), and interaction as the fixed effects. As for the random effects, they included the intercept for the subjects and the by-subject slope for the time duration and response side. This model fit the data significantly better than models that did not include the by-subject slope for the time duration, *χ*^2^(3) = 255.1, *p* < 0.001, or the by-subject slope for the response location, *χ*^2^(3) = 10.6, *p* = 0.014. The main effect of the time duration was significant, *F*(1, 30.9) = 172.267, *p* < 0.001, *η*^2^_*G*_ = 0.32, due to faster responses for the long (*M* = 390 ms, *SE* = 15.2) versus short (*M* = 527 ms, *SE* = 18.4) duration. Meanwhile, the main effect of the response side was not significant, *F*(1, 31.0) = 1.723, *p* = 0.199, *η*^2^_*G*_ ≈ 0. Importantly, the interaction was significant, *F*(1, 8093.9) = 6.237, *p* = 0.013, *η*^2^_*G*_ = 0.002, indicating the presence of a STEARC effect. Planned comparisons showed that for the short time duration, the responses were faster when provided with the left-side key (*M* = 520 ms, *SE* = 18.6) versus the right-side key (*M* = 533 ms, *SE* = 18.5; *p* = 0.012, *d* = -−0.17). Meanwhile, for the long time duration, no differences emerged for the responses associated with the left-side key (*M* = 391 ms, *SE* = 15.2) versus the right-side key (*M* = 389 ms, *SE* = 15.6; *p* = 0.717, *d* = 0.03; see Fig. 9).

### Discussion

The results confirmed the presence of a stimulus presentation time effect. More importantly, the temporal duration interacted with the response side, thus indicating the presence of a STEARC effect along the horizontal axis. The short temporal duration was responded to more quickly with the left-side key than with the right-side key, whereas no difference between the two keys emerged for the long temporal duration. This pattern of results provided a theoretical replication of the horizontal STEARC effect by adopting a task that closely resembled the one that Vallesi et al. (2008; Experiment 1) adopted. The next experiment tested the possibility to replicate the left-to-right horizontal STEARC effect when the two temporal durations employed in Experiments 1a/b are adopted (see also Experiment 3).

## Experiment 6

Everything was identical to Experiment 5 with only one exception: The visual target could last either 100 or 900 ms.

### Participants

Because the number of trials in this experiment was identical to that of Experiment 1a, data collection was stopped at *N* = 30 (*Mean age* = 24 years, *SD* = 2.65, 15 males) for convenience to achieve adequate statistical power. Three participants reported being left-handed, and the mean EHI was 69 (*SD* = 54.21, range: −100 to 100). More precisely, three participants had negative EHI scores (−63; −100; −100), and the remaining participants had positive EHI scores (from 63 to 100). All participants were naïve about the purpose of the study; they had normal correct-to-normal vision; and they provided written, informed consent that the local ethics committee approved (protocol number 1882). None of them had participated in the previous experiments.

### Apparatus

Everything was identical to Experiment 5.

### Procedure

Everything was identical to Experiment 1a, with the only exception being that manual responses were collected along the horizontal dimension as in Experiment 5.

### Results

The data were analyzed as in the previous experiments. The trials with anticipated responses (1.47% of the trials) or missed responses (0.33% of the trials) were removed. The trials with wrong responses (2.67% of the trials) were also removed and were not further analyzed due to their low percentage. The trials with correct responses and with RT 3 SD below or above the participants’ mean, computed separately for each experimental condition (1.716% of the trials), were considered to be outliers and removed.

The best model fitting data included the time duration (2: short vs. long), response side (2: left vs. right), and interaction as the fixed effects. As for the random effects, they included the intercept for the subjects and the by-subject slope for the time duration and response side. This model fit the data significantly better than models that did not include the by-subject slope for the time duration, *χ*^2^(3) = 647.9, *p* < 0.001, or the by-subject slope for the response location, *χ*^2^(3) = 55.19, *p* < 0.001. The main effect of the time duration was significant, *F*(1, 29.0) = 170.349, *p* < 0.001, *η*^2^_*G*_ = 0.43, due to the faster responses for the long (*M* = 323 ms, *SE* = 14.3) versus short (*M* = 491 ms, *SE* = 19.3) duration, as well as the main effect of the response side, *F*(1, 28.6) = 4.631, *p* = 0.04, *η*^2^_*G*_ = 0.003, due to the faster responses for the left-side response key (*M* = 401 ms, *SE* = 16.1) versus the right-side response key (*M* = 412 ms, *SE* = 15.7). Importantly, the interaction was also significant, *F*(1, 7564.9) = 5.045, *p* = 0.025, *η*^2^_*G*_ = 0.002, indicating the presence of a STEARC effect. Planned comparisons showed that for the short time duration, the responses were faster when provided with the left-side key (*M* = 483 ms, *SE* = 19.5) versus the right-side key (*M* = 499 ms, *SE* = 19.5; *p* = 0.004, *d* = -−0.20). Meanwhile, for the long time duration, no differences emerged for responses associated with the left-side key (*M* = 320 ms, *SE* = 15.0) and the right-side key (*M* = 325 ms, *SE* = 14.2; *p* = 0.331, *d* = −0.08; see Fig. 10).

### Discussion

The results were virtually identical as those observed in Experiment 5. Along with a reliable stimulus presentation time effect, the temporal duration interacted with the response side, meaning that responses for the short duration were faster when provided with the left-side key than with the right-side key, whereas no differences emerged for the long duration. This confirmed the presence of a left-to-right STEARC effect elicited by 100 and 900 ms temporal durations. In the next and final experiment, we tested whether a left-to-right STEARC could also be documented by using the same paradigm adopted in Experiment 2a, in which a denser temporal duration distribution was employed.

## Experiment 7

Everything was identical to Experiment 2a, with the only exception being that responses were collected through two horizontally placed response keys.

### Participants

Because the number of trials in this experiment was identical to that of Experiment 2a, data collection was stopped at *N* = 32 (*Mean age* = 24 years, *SD* = 7.26, 17 males) for convenience to achieve adequate statistical power. Three participants reported being left-handed, and the mean EHI was 66 (*SD* = 52.31, range: −100 to 100). More precisely, three participants had negative EHI scores (−25; −100; −100), and the remaining participants had positive EHI scores (from 13 to 100). All participants were naïve about the purpose of the study; they had normal correct-to-normal vision; and they provided written, informed consent approved by the local ethics committee (protocol number 1882). None of them had participated in the previous experiments.

### Apparatus

Everything was identical to both Experiments 5 and 6.

### Procedure

Everything was identical to Experiment 2a, with the only exception being that manual responses were collected along the horizontal dimension, such as in both Experiments 5 and 6.

### Results

The data were analyzed as in the previous experiments. The trials with anticipated responses (0.95% of the trials) or missed responses (0.35% of the trials) were removed. The trials with wrong responses (8.61% of the trials) were also removed and analyzed separately.^8^ The trials with correct responses and with RT 3 SD below or above the participants’ mean, computed separately for each experimental condition (1.719% of the trials), were considered to be outliers and removed.

The best model fitting data included the time duration (2: short vs. long), response side (2: left vs. right), and interaction as the fixed effects. As for the random effects, they included the intercept for subjects and the by-subject slope for the time duration and response side. This model fit the data significantly better than models that did not include the by-subject slope for the time duration, *χ*^2^(3) = 36.3, *p* < 0.001, or the by-subject slope for the response location, *χ*^2^(3) = 45.7, *p* < 0.001. The main effect of the time duration was significant, *F*(1, 29.5) = 602.788, *p* < 0.001, *η*^2^_*G*_ = 0.44, due to faster responses for the long (*M* = 386 ms, *SE* = 14.7) versus short (*M* = 557 ms, *SE* = 16.6) durations. Meanwhile, the main effect of the response side was not significant, *F*(1, 28.8) = 0.021, *p* = 0.887, *η*^2^_*G*_ ≈ 0. Importantly, the interaction was significant, *F*(1, 6259.4) = 29.994, *p* < 0.001, *η*^2^_*G*_ = 0.008, indicating the presence of a STEARC effect. Planned comparisons showed that for the short time duration, the responses were faster when provided with the left-side key (*M* = 546 ms, *SE* = 17.6) versus the right-side key (*M* = 568 ms, *SE* = 16.5; *p* = 0.012, *d* = -−0.31). Meanwhile, for the long time duration, the opposite occurred, and the responses were faster when provided with the right-side key (*M* = 376 ms, *SE* = 15.2) versus the left-side key (*M* = 395 ms, *SE* = 15.5; *p* = 0.022, *d* = -−0.30; see Fig. 11).

### Discussion

The results showed the presence of both a stimulus presentation time effect and an interaction between the temporal duration and response side, which confirmed the presence of a horizontal STEARC effect. Although short durations were responded to more quickly with the left-side key versus the right-side key, the opposite occurred for long durations (i.e., faster responses for the right-side key versus the left-side key). Overall, evidence of a left-to-right horizontal STEARC effect was also, therefore, provided in this case.

## Footnotes

1 The vertical direction is particularly salient when reading on a computer screen or on a poster placed vertically on a wall. In other circumstances (e.g., reading a sheet that lies on the surface of a table), reading follows a radial movement (i.e., from far to close relatively to the reader) rather than a genuine vertical movement. Nevertheless, it appears that, also in this case, the vertical direction is made salient by the fact that the text is read from top to bottom relatively to the spatial framework of the medium.

2 The total number of experimental trials was slightly smaller with respect to Experiments 1a/b to maintain a comparable time duration of the entire experimental procedure among experiments. Indeed, the overall temporal duration of the trials was longer in Experiments 2a/b than in Experiments 1a/b.

3 SNARC and SNARC-like effects are frequently tested by computing, for each stimulus, the mean RT difference between the two response locations, and then by fitting a linear model to these data with the target magnitude as a predictor. In this case, a negative correlation provides support to the existence of a SNARC or SNARC-like effect. However, when magnitude is task relevant (i.e., it is treated as an explicit dimension) as in Experiment 2a, the mean RT difference is a categorical function of magnitude, which implies the violation of one basic assumption of linear regression analysis (see Gevers et al., 2006). In this context, treating magnitude as a dichotomous categorical predictor (i.e., “shorter” vs. “longer”) appears to be more correct than treating it as a continuous predictor.

4 Wrong responses were analyzed through a mixed-effect logit model (Jaeger, 2008). The best model fitting data had the time duration (2: short vs. long), response location (2: top vs. bottom), and interaction as the fixed effects. As for the random effects, they included the intercept for the subjects and the by-subject slope for the time duration. This model fit the data significantly better than a model that included, as the random effects, only the intercept for the subjects, *χ*^2^(2) = 7.7, *p* = .021, and it did not fit the data significantly worse than a model that included the by-subject slope for the time duration and the response location, *χ*^2^(3) = 1.7, *p* = .631. The main effect of the time duration was significant, *b* = -.697, *SE* = .156, *p* < .001, due to more errors at the shorter versus longer time durations. No other significant results emerged (*p*s > .635).

5 Explorative analyses were also carried out only by considering the two temporal durations employed in Experiment 1a (i.e., 100 and 900 ms), in which a top-to-bottom STEARC effect emerged. The best model fitting data had the time duration (2: short vs. long), response location (2: top vs. bottom), and interaction as the fixed effects. As for the random effects, they included the intercept for the subjects and the by-subject slope for the time duration and response location. This model fit the data significantly better than a model that did not include the by-subject slope for the time duration, *χ*^2^(3) = 68.1, *p* < .001. It also tended to fit the data better than a model that did not include the by-subject slope for the response location, *χ*^2^(3) = 7.7, *p* = .053. Importantly, the time duration × response location was still significant (*p* = .021), indicating the presence of a STEARC effect. As for the 100-ms duration, the mean latencies were numerically smaller when provided with the bottom key (*M* = 515 ms, *SE* = 19.1) than with the top key (*M* = 526 ms, *SE* = 17.5), but the planned comparison was not significant (*p* = .265). As for the 900-ms duration, the mean latencies were numerically smaller when provided with the top key (*M* = 321 ms, *SE* = 16.4) than with the bottom key (*M* = 336 ms, *SE* = 18.7), and in this case, the planned comparison approached the canonical level of statistical significance, (*p* = .096). Overall, this pattern of results provides supporting evidence that the spatial representation of the 100 versus 900 ms temporal durations appeared to be reversed compared with what emerged in Experiment 1a.

6 In magnitude comparison tasks, regardless of the response location, the RTs tend to decrease linearly with absolute difference between the compared magnitudes. This is a well-known and widely replicated phenomenon called the *distance effect* (Moyer & Landauer, 1967), which has also been documented in a time duration comparison task (Beracci et al., 2021a, 2021b). However, in the present context, we refrain from testing the possible presence of a distance effect because of a confound between the distance effect and a stimulus presentation time effect. For instance, because of the distance effect, the responses to the 100 ms durations would be faster than the responses to the 400 ms durations (i.e., the distance between 100 ms and the 500 ms reference is larger than that between the latter and 400 ms). At the same time, according to a stimulus presentation time effect, the responses to the 100 ms durations would be longer than the responses to the 400 ms durations. In other words, in the case of durations shorter than the reference, the distance and the stimulus presentation time effects push in opposite directions (in the case of durations longer than the reference they push in the same direction). The tight interplay between the distance and the stimulus presentation time effects prevents a correct analysis of the distance effect in Experiment 2a, 4, and 7.

7 Wrong responses were analyzed as in Experiment 2a. The best model fitting data had the time duration (2: short vs. long), response location (2: top vs. bottom) and interaction as the fixed effects. As for the random effects, they included the intercept for the subjects and the by-subject slope for the time duration as the random effects. This model fit the data significantly better than a model that included, as the random effects, only the intercept for the subjects, *χ*^2^(2) = 76.4, *p* < .001, whereas more complex models failed to converge. No significant results emerged (*p*s > .533).
